# Memoir on the Discovery of Animal Magnetism

**Published:** 1839-04

**Authors:** 


### BRITISH AND FOREIGN MEDICAL REVIEW,

FOR APRIL, 1839.

### PART FIRST.

### Analytical and Critical Reviews.

### Art. I.

1. *Mémoire sur la Découverte du Magnétisme Animal.* Par A. Mesmer. —*Genève*, 1779. 12mo.

*Memoir on the Discovery of Animal Magnetism.* By A. Mesmer.—*Geneva*, 1779. 12mo.

2. *Ueber den thierischen Magnetismus in einem Briefe an Hoffmann,* (*mit Zusätzen.*) Von E. Gmelin, Physikus zu Heilbronn.—*Tübingen*, 1787. 8vo.

*On Animal Magnetism, in a Letter to Hoffmann,* (*with Appendices.*) By E. Gmelin, Physicus at Heilbronn.—*Tübingen*, 1787. 8vo.

3. *A Practical Display of the Philosophical System called Animal Magnetism.—London*, 1790. 4to.

4. ΑΣΚΛΗΠΙΕΙΟΝ. *Allgemeines medicinisch-chirurgisches Wochenblatt für alle Theile der Heilkunde und ihrer Hülfswissenschaften.—Berlin*, 1811. 104 Hefte.

ΑΣΚΛΗΠΙΕΙΟΝ. *General Medico-chirurgical Weekly Journal for all Branches of Medicine, and its collateral Sciences.—Berlin*, 1811. 104 Numbers.

5. *Versuch einer Darstellung des animalischen Magnetismus als Heilmittel.* Von C. F. A. Kluge.—*Berlin*, 1811. 8vo.

*Essay towards a History of Animal Magnetism as a Remedy.* By C. F. A. Kluge.—*Berlin*, [illegible]1. 8vo.

6. *Jahrbücher für den Lebensmagnetismus, oder Neues Ankläpieion. Allgemeines Zeitblatt für die gesammte Heilkunde nach den Grundsätzen des Mesmerismus; herausgegeben von* Dr. K. C. Wolfart, Königlich-Preuss. ord. Professor der Heilkunde an der Berliner Universität, Ritter des eisernen Kreuzes, 2ter Klasse, und des St. Annen-ordens, 2ter Klasse. 5 Bände.—*Berlin*, 1818-1823.

*Annals of Vital Magnetism, or New* Ασκληπιειον. *General Journal of Medical Science according to the Principles of Mesmerism; edited by* Dr. K. C. Wolfart, Royal-Prussian Professor of Medicine at the

University of Berlin, Knight of the Iron Cross (2d Class), and of the Order of St. Ann (2d Class). Five Volumes.—*Berlin*, 1818-1828.

7. *System des Tellurismus, oder thierischen Magnetismus. Ein Handbuch für Naturforscher und Aertze.* Von Dr. D. G. Kieser, Hofrath und Professor zu Jena. 2 Bande.—*Leipzig*, 1823.

*System of Tellurism, or Animal Magnetism: a Manual for Philosophers and Physicians.* By Dr. D. G. Kieser, Court-Counsellor and Professor at Jena. Two Vols.—*Leipsic*, 1823.

8. *Du Magnétisme Animal en France, et des Jugements qu'en ont portés les Sociétés savantes, avec le Texte des divers Rapports faites en* 1784, *par les Commissaires de l'Académie des Sciences de la Faculté, et de la Société Royale de Médecine, et une Analyse des dernières Séances de l'Académie Royale de Médecine, et du Rapport de* M. Husson; *suivi de Considérations sur l'Apparition de l'Extase, dans les Traitemens Magnétiques.* Par Alexandre Bertrand, Docteur en Médecine de la Faculté de Paris, Membre de la Société Royale Académique des Sciences.—*Paris*, 1826.

*On Animal Magnetism in France, and on the Judgments pronounced on it by the learned Societies, with the Text of the different Reports made in* 1784, *by the Commissioners of the Academy of Sciences, and of the Faculty, and of the Royal Society of Medicine, and an Analysis of the Report of* M. Husson; *followed by Considerations on the Delirium observed in Treatment by Magnetism.* By Alexander Bertrand, Doctor of Medicine of the University of Paris, Member of the Royal Academical Society of Sciences.—*Paris*, 1826.

9. *Die Seherin von Prevorst. Eröffnungen über das innere Leben des Menschen und über das Hereinragen einer Geisterwelt in die unsere. Mitgetheilt von* Justinus Kerner. *Zweite vermehrte und verbesserte Auflage.* 2 Bände.—*Stuttgart*, 1832.

*The Prophetess of Prevorst. Revelations on the inner Life of Man, and on a World of Spirits extending into our Sphere.* By Justinus Kerner. *Second, improved, and enlarged Edition.* Two Vols.—*Stuttgard*, 1832.

10. *Heilungen durch Animalischen Magnetismus bewirkt. Herausgegeben von* Dr. J. Bork, Physikus zu Altenschlirf in Oberhessen.—*Würzburg*, 1837.

*Cures performed by Animal Magnetism.* Edited by Dr. J. Bork, Physicus at Altenschlirf in Upper Hesse.—*Würzburg*, 1837.

11. *An Introduction to the Study of Animal Magnetism.* By the Baron Dupotet de Sennevoy.—*London*, 1838. 8vo.

12. *Fingerzeige Gottes, in göttlichen Offenbarungen für einer Somnambulen himmlisches und irdisches Heil.* (*Der Ertrag ist für einen wohlthätigen Zweck bestimmt.*)—*Weimar und Leipzig*, 1838.

*Hints from God, in Revelations for the Celestial and Terrestrial Salvation of a Somnambulist.* (*The profits to be devoted to charity.*)—*Weimar and Leipsic*, 1838.

13. *The Lancet and Medical Gazette.* Volumes for 1837 and 1838.—*London.*

The empire of medicine has just passed through one of those unaccountable paroxysms of credulity to which, from time to time, it seems ever to have been subject. Circumstances so plainly suspicious, and doctrines so obviously wild, that they seem only to require to be presented to any reasonable mind to be detected and rejected, are found, after certain or uncertain intervals, to excite the feelings and mislead the judgment of a very considerable proportion of the profession. Evidence, bold and presuming, pours in for a time on every side. The ignorant public, of the higher classes especially, catch the flame of enthusiasm; and grave medical authorities incline to relax their unbelieving frown, and smile on follies which are under powerful patronage. Whilst the reputation to be derived from science must be built up in solitude, with care and wasting toil, and many misgivings of the event, the bold necromancer comes, in these happy seasons, at once before the public; false lights blaze around; the music of flattery cheers him; the applause of the ignorant rewards him; the wealth of dupes enriches him. For a time reason is dethroned, and reflecting men repine under the government of misrule. In every house, in every society, at every dinner-party, they are shamed with a detail of wonders which they cannot deny and cannot equal; of facts which they have not witnessed, and of phenomena which they are almost reproached for not having produced. Their less scrupulous neighbours begin to be converted, begin to deal in magic themselves; and truth seems inclined to fly from the medical world. Nevertheless, there is comfort. The world is round, saith the proverb, and all things come to an end. Pass a few short months, and the delusion stands exposed: the actors are declared to be deceivers or deceived; the facts so lately boasted of are trampled upon with contempt, and the doctrines built upon them are laughed to scorn. The fashionable crowd flock to a new prima donna or to a watering-place doctor; and the half-converted physicians and surgeons never mention the subject more; for, although the folly will rise again, it will scarcely be in their time.

Such has been the past history, such has been the recent course of the doctrine and practice of Animal Magnetism. Often followed before, it had many times fallen into disrepute. Fostered by the Germans into life, and petted by the French into a sickly existence, our climate seemed always too cold for it. Many disguises it assumed; but the severity of physic abashed it, or the simplicity of philosophy put it out of countenance. Weak women and weak doctors went astray under its guidance, but scarcely any man of reputation had become a convert to it, and the public of England looked on the whole matter with incredulous and steady face. But *nemo omnibus horis;* and folly will have its turn. Even medicine is not always vigilant, and sometimes Æsculapius nods. In the nineteenth century, when knowledge is advancing in every direction, and when London abounds with scientific men, the *mime* called Animal Magnetism steps upon the metropolitan stage once more, waves his presuming wand, and performs with almost unbounded applause before crowded audiences. Women and children go to sleep at command, and wake only to sleights of hand. Their nervous system acquires intense sensibility. They become capable of impressions of sense in new modes. They obey volitions not reduced to words; they acquire knowledge which they never learnt; they understand what they were never taught. For them nothing is any longer obscure; to them nothing is unknown. They know all present things, and they prophesy events to come. And all this, and more, is produced by the influence of another person on the individual, which other person neither sleeps or wakes differently from common mortals; sees with his eyes, and feels with his hands like the vulgar; is not wiser than the philosophers; knows nothing of disease by mere intuition, but has studied medicine in the usual way; and who is no prophet, and cannot even foresee how all this necromancy, wrought by his unconscious hand, or effected by his suddenly omnipotent will, is to end at last.

There must surely be a sufficient number of persons of sane mind in the profession who have thought as we have thought, to exempt us from the suspicion of affecting wisdom after the fact, when we say that, from the first dawn of these diverting but degrading scenes to the last, from the first burst of blank surprise in the good unscientific public of this country, through all the phases of advancing credulity among the more scientific, down to the last complete and melancholy explosion, we have never varied from a most hearty, entire, and unconcealed disbelief of very nearly all the phenomena exhibited by all the patients, and related by all the practitioners, without exception. Several were plainly referrible to the boundless singularity of nervous and hysterical malady, and many were as evidently feigned. That there was gross deception somewhere, we were always sure: the only doubt we had was as to the precise point where the deception began. We beheld, always with astonishment, sometimes with concern, and sometimes with contempt, the credulity, real or pretended, of the magnetisers. We observed, with some little disgust, here and there a practitioner willing to become the *provincial* wonder, and only restrained by his prudence from declaring what a mixture of ignorance and cupidity prepared him to assert and to do. But, above all, we lamented to see the great delusion supported by one of the ablest physicians of this country, filling the most important chair in the largest medical school of the kingdom.

Of Dr. Elliotson's perfect honour and good faith we never entertained a doubt, in all the marvellous scenes performed in the North London Hospital. We were surprised to see him so content with insufficient evidence, and exhibiting so very marked an example of a total want of cautious observation and philosophical reflection. We blushed to read of lords and gentlemen chuckling over the discovery that they could communicate that intelligence to others which they so plainly wanted themselves; and we did not feel surprise when, at a watering-place hotel dinner, soon afterwards, we found one of these supposed patrons of science (although not, we believe, a magnetiser,) declaring that the fame of a celebrated watering-place doctor in England made the physicians of London and Edinburgh sick at heart. The scenes exhibiting in London left us without courage to controvert this graceless libel on a profession once deemed liberal and learned. At length came the *dénouement;* and nervous women and children, and doctors and noblemen, disappeared like so many shadows; and country doctors assumed an air of unshaken wisdom, and were laudably anxious to bury the whole subject in oblivion. Impossible as it is to preserve an entirely serious tone when speaking of these things, they are really of a most melancholy nature. Considering the high sanction which even a temporary belief in the powers of animal magnetism has obtained in this country, we look upon its recent rise and progress, and its abrupt and shameful fall, as powerfully calculated to degrade a profession which is certainly, for other reasons, not rising in public estimation.

To devote an article to the consideration of animal magnetism, now that the English practitioners are one and all ashamed of its name, would be a work of supererogation, if the delusion, unabashed, were not yet parading itself over some parts of the continent; and if its return to these shores, and to our own hospitals and colleges, at any future period, were quite out of the question. But if we can quicken its decline where it now reigns in the hearts of nervous proselytes and dreaming physicians, or can assist in forming a barrier against a probable revisitation of it, we shall not think the otherwise more than due attention we have given to the wild productions which treat of it entirely thrown away. Independently, however, of all these considerations, the history of the phenomena resulting from the various forms and practices of what has been called animal magnetism, whether these phenomena be considered as physical or moral, or partly both, are among the most curious in the whole history of man. The cool effrontery and undoubted skill of some of the magnetisers, and the *bonhomie* of others; the subjugation of the bodies and souls of the magnetised; the puzzled attitude of learned societies; the vain authority of the police; and the gradual dissipation of the whole subject again and again from the public mind, magnetisers and magnetisees disappearing like the actors in a play, are of a nature highly to amuse, and not a little to instruct, all those who love to regard human beings (who advance high claims to the profession of reason), in various points of view.

ANTHONY MESMER, who, in the "Annals of Vital Magnetism," edited by a Professor of the University of Berlin, is gravely stated to have been the greatest man that the world ever produced—was born, we know not where or when: according to some, he first saw the light at Vienna, in 1740; according to others, he was born at Merseburg in Swabia, in 1734; whilst others again assert, that Switzerland was the land of his birth, but leave us in the dark as to the time and exact place of the first appearance on the stage of life, of "this noble being, this faithful Christian, this Theologian, Philosopher, Jurist, Mathematician, Chemist, and Physician," (for in all these characters is he eulogized by one of his Prussian admirers, Dr. von Voss.*) His first work, a dissertation on the influence of the planets on the human body, was published in 1766. It was not, however, till 1772, when he began to turn his theories to a practical account, that he produced any sensation in the public at large. His announcement of the discovery of a universal fluid, "the immediate agent of all the phenomena of nature, in which life originates, and by which it is preserved," might have shared the fate of the thousand crude and fantastic theories which are continually floating and bursting on the surface of Time's restless stream, had he not boldly declared himself divinely empowered to regulate the operations of this fluid, to guide its

* Wolfart's Annals, vol. i. part i. p. 83.

currents in healthy channels, and obliterate, by its means, every track of disease. Goethe has laid it down as a precept to the man whose vocation it is to gull the public, that he should never be afraid of making too preposterous demands on its credulity; should never trouble himself by forming delicate and ingenious devices for ensnaring its belief; but should at once "commit downright daylight depredations" on its faith. Mesmer needed not this formal precept; he instinctively obeyed it. He commenced his voyage of wonders by launching this simple assertion, "There is one health, one disease, one remedy, and one physician—and that physician am I." Such a declaration was not immediately successful; some of its hearers expressed doubts as to the sanity of its propounder; others condescended to oppose themselves formally to its reception; others disregarded it; but Mesmer persevered, undoubting and undismayed, and shortly saw the time arrive when his modest pretension not only filled his coffers with fees, and induced the French government to sue him to accept a pension and a palace, but actually won for him the sum of 340,000 francs in hard cash, which were paid him by his devoted disciples, as the price of initiation into those secrets of nature, of which he boasted himself to be the sole discoverer and depositary. It was by the magnet that he first pretended to regulate the distribution of the universal fluid, which was to fill his particular pocket; and the magnets for his experiments were furnished him by Father Hell, a professor of astronomy at Vienna, who, when he announced the curative effects which they produced in his hands, claimed the merit of the discovery, and, setting aside his theory, explained their therapeutical properties by their form and construction. A very pretty quarrel now ensued between these philosophers, Hell accusing Mesmer of stealing his magnetical discoveries, and Mesmer reproaching Hell with attributing to his magnet, and indeed with appropriating, the virtues of the new Mesmerian universal fluid: the advantage, such as it was, of the squabble, remained with the holy Father, who it seems was better known and more respected than his antagonist. Mesmer's ready talent, however, soon avenged him on the astronomer, and gained him more ground than he had previously lost: he shortly promulgated the discovery, that the new universal fluid, which, though it pervaded all space and had existed from all time, he regarded, it appears, quite as his own property—as a kind of estate entailed on him and his family—was not exclusively affected by the contemptible magnets of a priestly star-gazer, but under his hands could be concentrated in and liberated from any substance he pleased, as paper, bread, wool, silk, leather, &c.: he even asserted that he could charge jars with it, and of course discharge them at pleasure. Health he now defined to be a regular and harmonious distribution of this fluid throughout the body: in its deficiencies and anomalous currents he found the cause of every disease; and his treatment consisted in setting up what he called an artificial *tide*, or ebb and flow, throughout the system, until the fluid reached a healthy level, or resumed a smooth, unruffled course, forming neither broads nor rapids, and resembling rather the stream of a canal than that of a romantic river.

By means of a theory crude and absurd as the above did Mesmer not only enrich himself, as we have already said, but actually instituted a school, not composed solely of lay enthusiasts and mere fanatics, but of learned professors and of medical practitioners; who, far from disdaining his protection or seeking to disavow or modify any of his doctrines, declared themselves unreservedly his disciples, spoke of him with affectionate veneration and even awe, holding him up indeed in their organ—the *Annals of Vital Magnetism*—as the divinest of philosophers, and greatest of physicians.

We find Mesmer in 1775 and 1776, travelling in Bavaria and Switzerland, visiting the hospitals, and creating a great sensation by his mysterious manipulations, and by the novel effects which they frequently produced on hysterical and half-witted females. On his return to the Austrian capital, he succeeded in rousing the attention of the public to his proceedings, by affirming, that he had cured of complete blindness a celebrated singer, Mlle. Paradis, who had been for ten years unsuccessfully treated by Herr von Störk, physician to the court. It would almost appear, that there was some collusion on this occasion between Mesmer and his patient: at any rate, there was considerable difficulty in ascertaining whether she had been relieved by him or not; some scandalous scenes took place, and at length she was got out of his hands and found to be as blind as ever, although he had the assurance to thank heaven that he had been able to restore her sight, and that the disturbances which his adversaries had excited, had not deprived her of the beneficial results of his treatment. This whole exploit redounded so little to his credit, that he found it necessary precipitately to quit Vienna; according to some, he was ordered by the Empress to leave the capital at twenty-four hours' notice.

Shortly afterwards we find Mesmer at Paris, where his fame had already preceded him. The state of French society was at that time particularly favorable to the reception of his marvellous doctrines and mysterious practice; a feverish excitement prevailed, which, instead of encouraging enquiry into the social evils in which it originated, induced men to suppress all anxious presentiment, and to defer serious thought, so long as they could find frivolous disputants or marvel-mongers, who could divert their attention from the dangers with which they were threatened. Matters of the least possible moment were now sufficient violently to agitate the public. The heads of different schools of music found partisans, who attacked each other with all the zeal and bitterness of political controversy. A vague commotion pervading all classes of society was the forerunner of the terrible storm which was shortly to convulse and to confound them. Amongst the most ardent supporters of Mesmer at Paris were two men who were afterwards to play conspicuous parts in the revolutionary drama, La Fayette and D'Epremenil. Mesmer was neither slow in perceiving the signs of the times, which were capable of being turned to his advantage, nor backward in making the best use of them. He at once declared himself "*un homme de génie et un bienfaiteur de la race humaine*," and fixed the price of his medical attendance at ten louis d'ors per month. His subsequent proceedings corresponded admirably to these preliminary measures. Feeling that the enthusiasm which he excited in the public would soon die away if it did not encounter ardent adversaries, he provoked the learned societies of the French capital to enter the lists against him, and raised a storm of opposition which had the effect of attaching his disciples devotedly and indissolubly to his cause, and of enabling him to appear in the character of a martyr sacrificed at the altar of falsehood. The infatuation, under such circumstances, of the vulgar, the idle, and the imbecile, may be easily accounted for; but we cannot suppress our astonishment when we find men of learning, reputation, and real rank in society ranged on the side of an impudent impostor. Deslon, physician to the Count d'Artois, was one of the first who joined him: among other subsequent disciples came Court de Gibelin, author of "Le Monde primitif," who addressed a long appeal to the public in his favour, in which he speaks of him in the most extravagant terms. He details his own cure by magnetism, and that of a Mademoiselle de Berlancourt, who is represented as saying,

> Infans, cæca, trahens gressum, te, Mesmere, posco
> Verba, pedes, oculos: Ambulo, cerno, loquor!

The recovery of this young lady is authenticated by her uncle, M. Michel, by the bishop of Beauvais, by a physician, three surgeons, and, to give weight and force to the testimony, by nearly a whole regiment of soldiers, who were quartered in the town in which she lived. "To whom," says Gibelin, "do I, the author of 'Le Monde primitif,' owe my existence? To Mesmer, the saviour of men!" This individual was no vulgar dupe; he was a royal censor, honorary president of the Parisian museum, and member of several academies. Shortly after the publication of his appeal, he died whilst under a course of magnetic treatment, and instantly the following paragraph went the round of the anti-Mesmeric journals: "M. Count de Gibelin vient de mourir, gueri par le magnétisme animal." An examination, however, proved that he had long been labouring under organic disease.

The mystery in which Mesmer enveloped his practice was well calculated to sustain the interest which his first appearance had excited. For the treatment of patients he fitted up a spacious apartment, in which a perpetual twilight was ingeniously maintained; the air was impregnated with the most exquisite perfumes, and every now and then strains of music (in which science he was a proficient) stole on the ear of the languishing and susceptible invalid. In the middle of the room was placed a circular vessel (*le baquet*), containing bottles of water which had previously been magnetised: the lid was perforated for a number of curved and moveable iron rods, which branched off in every direction, and the extremities of which were grasped by the patients, who thus stood in a circle, sometimes holding each other by the hand, and forming what was called "the chain." It would seem that Mesmer was not present during the first stage of the magnetic operation; he reserved his interference till it had brought about a crisis, when, arrayed like a magus, he stalked in, waving a mysterious wand, by the motions of which he decided the catastrophe of each individual case. On first standing round the baquet, the most various symptoms were developed: some of the patients appeared apathetic or indifferent; others were slightly convulsed; as the excitement increased, particular individuals were observed to rush towards each other, with smiles and expressions of affection, seeking mutually to mitigate the violence of their crises: as soon as the disorder and confusion were at their height, the majestic figure of the great magnetist was observed gliding towards the scene, his wand upraised to stay the storm ere it reached an uncontrollable pitch. It is said in the Biographie Universelle, that he had confederates amongst his patients, who set the example of blind obedience and resignation to his wand and will immediately on his appearance, and whose tacit cooperation cost him more than 100,000 francs. Certain it is that no sooner did he approach the baquet, than the tumult began to subside; the patients turned their loving regards from each other to their common magnetiser, who now put his finishing hand on the magnetic process, giving it such a termination as seemed to him best. Where the case was particularly interesting, or was capable of being made particularly profitable, the magnetiser performed the whole operation himself; the position on such occasions being "forehead to forehead, and foot to foot." When the patients laboured under very violent or unmanageable crises, they were carried into a chamber fitted up with mattresses and cushions for their reception, where, it appears, they were left to fight or kick it out as they best could. Young and attractive females the magnetiser generally preferred subjecting to the immediate or personal process; and the effects which it produced upon them are detailed in the following passage of the Secret Report of the Commissioners appointed, in 1784, to enquire into animal magnetism.

"The greater number of women who are magnetised are not really ill; they come for amusement, or for want of something better to do; others, who may be slightly indisposed, are not less fresh and vigorous: their senses are not impaired, their youth has all its sensibility. Continued proximity, contact, the communication of bodily warmth, and the mingling of glances, are the well-known ways and means by which nature always prepares to effect a community of sensations and affections. The man who magnetises has generally the knees of his female patient enclosed between his own: all the inferior parts of the body are consequently in contact. The hand is applied to the hypochondric regions, and sometimes over the ovaries. Touch is exercised over a large extent of surface, and in the neighbourhood of the most sensitive parts of the body. Frequently the man, having his left hand thus applied in front of the woman, passes his right hand behind her; each then moves towards the other, in order to favour a double pressure; they approach as closely as possible; face touches face; their breath mingles; all physical impressions are instantaneously communicated, and the reciprocal attraction of the sexes acts, of course, with all its force. It is not extraordinary that the senses are now kindled; the imagination throws the whole system into disorder, suspends the judgment, and precludes reflection: the women can often give no account of what they experience, and are ignorant of the state in which they are. . . . When this state of crisis approaches, the visage fires by degrees, and the eyes light up with desire. The woman bends her head, and seeks to hide her forehead and eyes with her hand; a natural shame prompts her instinctively to conceal herself. Nevertheless, the crisis is continued, and the eye is troubled; an equivocal sign of the total disorder of the senses. . . . The eyelids now become moist; the breathing hurried and irregular; the bosom heaves violently and rapidly, and convulsions and sudden twitchings take place in particular limbs, and sometimes all over the body. In lively and sensitive women, the last stage, the most agreeable termination of their emotions, is often a convulsion. To this state succeed languor, depression, and a sort of slumber of the senses; a necessary repose after violent agitation."

The commissioners do not appear to have regarded even these scenes as sufficient to determine their opinion. With Franklin, Bory, and Lavoisier at their head, they send to consult with the lieutenant-general of police. This personage arrives, and assuming, as we may suppose, all his official severity, formally addresses Dr. Deslon as follows: "In my capacity of lieutenant-general of police, I demand of you whether, when a woman has been magnetized, and is in a crisis, it would not be very easy to seduce her?" To this simple question Dr. Deslon returned the very simple answer, "that it certainly would." Upon which the commissioners take their leave of him, and concoct their "Secret Report," in which they give it as their decided opinion that the practice of animal magnetism is immoral, and ought to be prohibited.

The personal career of Mesmer comprises, for some time, the history of magnetism. It appears that, during the first years which he spent at Paris, he received immense sums from his patients: he afterwards confessed that, in 1784, his income was not less than 400,000 francs. His reputation at length was such, that the government actually offered him 20,000 francs per annum, and 10,000 francs to build an establishment for pupils and patients, on condition that he should remain in France, and allow three persons appointed by the government to watch, and report on, his proceedings: nor were the advantages thus offered him to be annulled, should even the report of these commissioners prove altogether unfavorable to him. These terms were far from satisfying Mesmer, and he promptly rejected them; hoping, probably, by holding out, to obtain others much more advantageous. "The propositions made me," he said, "seem to me to err in having my pecuniary interest, and not the importance of my discovery, for their principal object. If my discovery is not credited, it is evidently wrong to offer me for it a pension of 20,000 francs annually: *if it is credited, the fate of humanity ought not to be sacrificed to the self-love of a few 'savants.'*" He means here his enemies of the scientific societies, with whom he was continually embroiled, and whose opposition drove him at one time to such an extremity that he declared that "he thought for three months without the aid of words." In his "Précis historique de la Découverte du Magnétisme Animal," he opens his mind still more freely to the government, stating "that he had always expected a recompense worthy of the French nation and of the monarch who governed it; that it was delicacy alone which indisposed him to receive a sum to defray the expenses of an establishment, and that he wished to hold, as a direct gift from the munificence of the state, a *territorial possession.*" "I well know," he adds, "that the sum which I demand is considerable; but I also know that my discovery is beyond all price. In the eyes of your majesty," he continues, addressing the king, "*four or five hundred thousand francs, more or less,* well employed, are nothing: the happiness of your people is all. My discovery ought to be received, and I recompensed, with a munificence worthy of the monarch to whom I shall attach myself." This was written in 1784, yet, so late as 1818, we find Wolfart, professor of medicine in the university of Berlin, exclaiming, with indignation, "And Mesmer is accused of having been avaricious!"* The French government, weak as it was at that time, appears to have known the value of "*four or five hundred thousand francs, more or less,*" (as well it might, considering the state of its exchequer,) and accordingly Mesmer's tempting bait was not ho-

* Annals of Vital Magnetism, vol. i. part i. p. 12.

noured by even a single nibble. In a pet, the philosopher determined to withdraw from ungrateful France, and leave her unmesmerised sons once more a prey to every disease: he accordingly retired to Spa, where he was shortly followed by numbers of his disciples. In his absence, Deslon, his early convert, announced to the Faculty of Medicine at Paris that he was acquainted with Mesmer's discovery; and, in effect, he fairly stepped into the latter's shoes, and carried on his course of treatment. As soon as this news reached Spa, the persecuted fugitive was seized with an indignation which knew no bounds: he at first appeared crushed by the weight of this new misfortune; then he exclaimed repeatedly that he was betrayed and ruined; that he had been perfidiously robbed of the fruit of his long watchings, of his precious discovery, his sacred property.* It was now that his disciples, to console him in his tribulation, and recompense in some measure his merits, as well as at the same time to secure for themselves what they imagined was an inestimable prize, determined to raise, by contributions of not less than 100 louis d'ors each, the sum of 240,000 francs, to purchase from Mesmer the knowledge of his theory and practice. A subscription was instantly entered into, and 90,000 francs more than was originally proposed were poured into the pocket of the philosopher. As soon as he had instructed his subscribers in such of his practices as he found it convenient to make them acquainted with, he forbade them to make public, or indeed to communicate to any person, anything he had taught them; and, when they refused to obey this preposterous injunction, he accused them of wronging and swindling him, and affirmed that they had all entered into a solemn engagement not to reveal his mystery; an affirmation which was utterly false.† After a great deal of wrangling and invective, he proposed that subscriptions, to which no person was to contribute less than fifty louis, should be entered into in the principal cities of France: half of the produce of these subscriptions to accrue to him, and the other half to go towards diffusing a knowledge of his discovery. His disciples, however, refused to listen to such terms; and Mesmer, totally disgusted with the treatment he had received at the hands of an ungrateful world, took a final leave of his adopted country, complaining, wherever he went, that his discovery had been wrested from him, contrary to all justice, and in violation of all the laws of honour. On the other hand, the purchasers of his secret were equally dissatisfied with his conduct: one of them, M. de Bergasse, publicly attacked him; and another, Berthollet, the celebrated physician to the Duke of Orleans, at first a regular disciple of Mesmer, was so thoroughly undeceived by the initiatory process (price 100 louis d'ors), that he published a declaration, utterly condemnatory of the whole Mesmerian system, and terminating thus: "I declare, in short, that I regard the doctrine of animal magnetism, and the practice to which it serves as a foundation, as perfectly chimerical."

Notwithstanding the checks which his own sordid conduct and the defection of some of his more noted followers gave to the system of Mesmer, such was the infatuation of the public and the morbid excitement of the times, that it still continued to flourish. Modified by

* Bertrand, p. 48.

† For the particulars of this dispute, see Bertrand, p. 52.

Puysegur, it found refuge in somnambulism from the blows aimed at it by the commissioners of the Faculty and of the Academy, which were tolerably effective in demolishing its early pretensions. France, as might be expected, was the hotbed where it principally flourished; but stray seeds were blown over to Germany, where, in 1787, we find Gmelin magnetising, or, as he calls it, "manipulating" a host of women at Heilbronn and in its neighbourhood. Nor did the Straits of Dover present any effectual barrier to its propagation into England, where, in 1788, Maineduc, a pupil of Deslon, is represented by Hannah More as making 100,000*l.* by its practice;* a success which fully explains the recent invasion of this country by the Baron Dupotet de Sennevoy. About the period in question, an immense sensation was created by animal magnetism, in the provinces as well as in London; but it was merely ephemeral, and scarcely any records have been preserved of it. A person called Holloway is said to have realized a considerable fortune by giving lectures on the subject, to which the admission-fee was five guineas. But the most extrordinary performers with the animal magnet were a Mr. and Mrs. de Lauterbourg, residing at Hammersmith terrace, of whose wonderous cures a Mrs. Pratt, 41, Portland place, Marylebone, advised the Archbishop of Canterbury, in a letter dated June 21st, 1789. After this letter comes a list of cures, and before it is a paragraph, informing the reader that the publication has been issued quite contrary to the will of the divine M. de Lauterbourg; but that the philanthropic authoress prefers risking his displeasure to hiding any longer his miraculous powers from the public. De Lauterbourg's house at Hammersmith was beset by crowds seeking admission, either out of curiosity or for medical relief; and entrance tickets, which he gave away, were sold for from one to three guineas each. Mrs. Pratt begins her address to the archbishop by announcing that both Mr. and Mrs. L. had been rendered by God "proper recipients to receive divine manuductions." She proposes a public thanksgiving for the blessings they dispense, and a form of prayer for their continuance. "Let us join," she says, "in prayer and praise to have this most glorious blessing continued, lest our candlestick be removed from us, which I most ardently pray the Lord Jehovah to avert." Mr. and Mrs. de Lauterbourg cured two thousand people in six months; they were not at all particular in selecting cases, restoring indiscriminately the "deaf, dumb, lame, halt, and blind," young men dying of scrofula, and women possessed with devils. Their method of treatment was very mild, as we learn from the following case, published by Mrs. Pratt: "Mrs. Hook, stable-yard, St. James's, has two daughters, born deaf and dumb. She waited on Mrs. de L., who looked at them with an eye of benignity, and healed them. (I heard them both speak.")

About 1790, various tracts on magnetism were published in London, the authors of which, after roundly abusing each other, asserted, each of them, that he alone was the true prophet, and that all the rest were unmagnetised heretics: they laid down their premises thus; "It is now generally admitted that there is a plenum or universal fluid," &c. &c. Shortly afterwards, tracts and tractors, magnetisers and magnetised, disappear altogether from the scene, and England resumes its wonted

* Dupotet, p. 319.

quiet.—Such was the farce of animal magnetism as first acted in this country: after having caused great excitement, it sank rapidly into forgetfulness, there to remain till revived in our days by a company of actors far more philosophic, profound, and learned, doubtless, than their rude predecessors; equal to them, certainly, in every histrionic qualification, but unhappily even less successful with the public.

Shortly after Mesmer had retired from France in disgust, the French revolution broke out, and the universal fluid was lost sight of in the universal commotion which ensued: what its venerable proprietor did with himself and it during the troublous times which quickly followed his second exile, we have not been able to discover. Shortly after the commencement of the present century, however, we find him and his tub in Switzerland, busily engaged, though, of course, on a very limited scale. He died in comparative obscurity, on the 5th of March, 1815. Dr. Hirzel, of Gottlieben, thus announces his decease to Wolfart: "Yesterday morning, at eleven o'clock, it was my destiny to close the eyes of the greatest man that the world ever produced."* His funeral sermon, a "remarkable and touching" composition, was preached by a young priest whom he had converted to magnetism, and who was indebted to him for many important revelations concerning "religion and nature." The Berlin professor of medicine celebrated his apotheosis in lofty rhyme.

If we were not treating exclusively of animal magnetism, we should be justified in maintaining that here human folly and credulity must have attained their acme; but it is the peculiarity of this interesting subject that, on examining it, we find no limit to absurdity; no climax beyond which some one does not leap into still more mystic regions, beyond whom, again, we descry some still bolder head of somnambuliser or somnambulist in the distance.

Wolfart's Annals contain dull letters from Mesmer, written in bad German. In No. 283 and No. 284 of the "Morgenblatt" are extracts from a lecture on the character of Mesmer, delivered at Zurich, by Dr. Egg. von Ellikon, who made his acquaintance in 1804, and who was afterwards frequently in communication with him: this critic is evidently impartial, and we shall fill the very limited space which we can afford further to devote to the great father of animal magnetism, with his observations. He describes him as an old man of a venerable appearance, talkative (especially when the subject of conversation was his own merits and discoveries), and assuming towards his patients, and indeed whenever the practice of magnetism was mentioned, an air of mystery which was altogether repulsive. He was accustomed to speak with the greatest contempt of those who differed from or opposed him, and was never tired of sounding his own praise, and of dwelling on the benefit which his magnetic discoveries had conferred on mankind. In his sitting room hung a painting in which he was represented as the good genius of the world, celebrating the triumph of animal magnetism over medical science. He was in the habit of presenting those who made his acquaintance with a print of himself, under which were some French verses, extolling him in the most fulsome terms. When his discoveries were the subject of discussion, he invariably finished it by a violent tirade against the ingrati-

* Wolfart's Annals, vol. i. part i. p. 13.

tude of the world, and the persecution he had suffered from the medical profession: medical men he called poisoners, and all their drugs, poisons; against all modern magnetisers, too, he was highly incensed, accusing them either of not having been able to understand him, through stupidity, or of having betrayed him. Bitter were his complaints that the somnambulisers were ruining the science, and doing more harm to the good cause than the most deadly blows of its most vehement adversaries. He said once to Dr. E., "It is true I am old, and may yet live many years; but I know, for certain, that I should live ten years longer than I now shall do, if a surgeon had not once bled me when I was young." Midwives and man-midwives he classed together under the name of privileged murderers of mankind. The tying of the umbilical cord he held to be the cause of the small-pox and of all hepatic diseases, under which he classed almost every chronic malady. Beyond his own theory and pretended discovery, he knew and cared about nothing; his reading was confined to two or three newspapers; of the progress of science he was altogether ignorant; and even his political opinions, strange to say, were modified by his peculiar views, and he actually advocated a political revolution and reorganization on magnetical principles. He pretended that he could only think in French, and that he translated from the French whatever he wrote in German. When Dr. E. first became acquainted with Mesmer, he was doubtful what to think of the effects of animal magnetism upon the human system. Mesmer, of course, laboured hard to win over the waverer; but the latter is obliged to confess that, the more he now saw, the less he believed: it seems he would have been a convert had not Mesmer prevented him. As the two were one day walking together, Dr. E. asked the philosopher why he always ordered his patients to bathe in river water, and not in spring water? The latter answered, "Because river water is exposed to the sun's rays." "I know," observed the other, "that river water is sometimes warmed by the sun, but not so much so that you are not frequently obliged to warm it still more, and therefore I do not see why warm spring water should not often be preferable. "Dear doctor, the cause why all water which is exposed to the rays of the sun is superior to all other water is because it is magnetised. Twenty years ago I magnetised the sun, and since that time," &c.———

Having disposed, we hope conclusively, of Mesmer, we now come to speak more particularly of the nature and progress of the system which he engendered. To treat first, then, of the theory of Mesmerism. The founder of the system pretended, as we have seen, that the entire universe was plunged, as it were, into a vast ocean of fluid, which penetrates it throughout, and produces in it all the phenomena which we observed around us. This fluid, he continues, is the medium of an influence which the heavenly bodies, the earth, and animated nature continually exercise upon each other. The human body has properties analogous to those of the magnet; it has poles "*également divers et opposées;*" and it can operate upon the universal fluid. "By means of magnetism, the physician is acquainted with the state of health of every individual, and perceives with certainty the origin, nature, and progress of the most complicated maladies; it prevents their increase and effects their cure, without exposing the patient, of whatever age, temperament, or sex, to any danger. Nature offers in magnetism a universal method for curing and preserving mankind."* This creed was fully adopted and professed by Wolfart, Ziermann, and indeed the whole school of Berlin, which, as we have given the reader numerous opportunities of seeing, speaks of Mesmer as of a being whose dogmata are sacred, and whose very hints and incidental observations (however apparently trifling or contradictory) deserve to be treasured up as golden rules. It was according to this system that Wolfart, Royal-Prussian professor of medicine in the university of Berlin, and knight of the iron cross (second class), treated in the Mesmerian hospital, and in his private practice, in the year 1820, 1428 patients afflicted with all kinds of maladies, mental and corporeal, external and internal, febrile and inflammatory;† of whom, according to his own report, 632 recovered, 599 improved, 140 remained uncertain as to whether they were cured or no, 24 got worse, and 13 died. Of the other twenty no account is given. Wolfart, though a blind follower of Mesmer, does not content himself with the letter of the law as laid down by the latter, but seizes its spirit, and works it into aphorisms and axioms of his own, from which we may learn more closely the view he takes of the Mesmerian philosophy and practice, and of which the following are specimens.

"Mesmerian or vital magnetism, is neither a medium nor a matter, nor a power of itself, but is the nominal definition of the relative changes taking place in the physical and psychical or moral world."‡ Now this means, if it means anything, that no act, motion, or change whatever takes place except by means of Mesmerism. It asserts, as plainly as an abstract position can, that Lisbon was swallowed up by Mesmerism; that a comet clashing against a planet is Mesmerism; that the battle of Waterloo was Mesmerism; that Shakspeare mesmerised when producing his immortal poems; in short, that the first great Mesmer was the Creator of the universe. But it has not been left to the adversaries of magnetism to draw these deductions from the position above laid down.

In a work entitled "Considerations on Animal Magnetism, especially in regard to numerous Phenomena of the Past and Present connected with it; by J. A. L. Richter, con-rector of the principal ducal school at Dessau," published at Leipsic in 1817, we find the author thus stating the grand object of his study: "It consists in nothing less than in the solution of many enigmas of human existence, and particularly of the enigmas of Christianity, on the obscure and mystic parts of which a light is now thrown (by magnetism, of course,) which permits us to gaze clearly on the secrets of the mystery." He then proceeds to state that all the miracles of the New Testament were performed by means of animal magnetism; which is also accountable for all the wonders and witchcraft of the middle ages, and which he finally declares to be no other than Omnipotence itself. "Magnetic instinct" is the principle by which he explains the creation and conservation of the world, and amongst those who have conspicuously manifested it he enumerates, almost in the same breath, Adam, Müller, Madame von Krüdener, St. Paul, Luther,

* Mesmer sur la Découverte du Magnétisme Animal, pp. 6 and 74.

† Annals, vol. v. part i. p. 38.

‡ Annals, vol. i. part ii. p. 49.

a number of old women, Jesus Christ, and, finally, the Almighty. This author, we must not forget to remark, is repudiated by the pure Mesmerians, and belongs to the new and improved school of magnetism, that of the somnambulisers: we already begin to see that Mesmer is respectable and innocent compared to some of the disciples of this latter sect.

We must content ourselves with giving one more only of these aphorisms of Wolfart; a rich specimen, which we shall leave without note or comment.

"When the vital dance of the viscera flags, we must lend it a helping hand. We must strike up, and play vigorously, joyously, and in elevating harmony: then the organs which were fatigued, or disordered, or out of tune, will begin to dance regularly in intertwining mazes, until at length they will sing to themselves the appropriate rhythm, without requiring the aid of our medical music. But, were we to fiddle unmelodiously, or too violently, the viscera would remain deaf and unmoved in their places, or would fly the scene, and there would be no dancing. The best medicine of the ordinary kind can only strike up a tune, and that truly is much; but magnetic medicine can not only strike up a tune, it can lead and join the dance; and that is much more."*

According to Mesmer and his more faithful followers, magnetism only produces evident symptoms on those whose health is deranged, and often not even upon them; though this is quite contrary to the doctrine of more modern practitioners, who profess to be able to mesmerise where Mesmer himself would be powerless, and who magnetise indiscriminately both those in and those out of health.† Dr. Ziermann, an excellent writer, as far as style and method are concerned, in Wolfart's Annals, states that a gradually diminishing influence of magnetism upon the patient is the best indication of his improvement; that the patient, in fact, is not cured so long as he is magnetisable. He expressly says that, as a general rule, the healthy subject is not capable of being magnetised; and he adds, what is doubtless very true, that the attempt to mesmerise a girl who is in health may often have the effect of precipitating her into a disease.‡ The hopes which some of Mesmer's followers entertain of the magnificent results of his system are the best indication of the exalted opinion they hold of its essential nature and practical bearings. Says Dr. Riecke,§ of Stuttgard, "I am much too feeble to have even a presentiment of the tremendous results which must necessarily flow from Mesmerism, much less am I capable of describing them." He states that he formerly wandered in a medical morass, led by an *ignis fatuus;* that he sought for a guiding star, and "at length with shouts of joy hailed it beaming bright on the horizon, in the shape of Mesmerism, which is to remedy all our defects." The first thing which the new star promises is to blight all apothecaries, and close all apothecaries' shops. Dr. Riecke never enters one of the latter without a secret shudder, and without fancying that he is in a witch's cave. The only difference, he says, between the ancient and modern apothecaries is, that the former sold poison to

* See Wolfart's Annals, vol. ii. part ii. p. 29.

† Dupotet says, "It is a great mistake to suppose that magnetism only acts on weak and nervous persons. I have often magnetised men of the most robust habit, and produced on them more remarkable effects than on persons of less robust constitutions. No one should ever be considered an unfit subject." (p. 154.)

‡ Annals, vol. v. part ii. p. 5.

§ Annals, vol. ii. part ii. p. 2.

those who asked for it, and that the latter cram it down the throats of those who ask for any thing but it. Mesmer himself was not more violent in his language towards the medical profession than this, his professional disciple, who exclaims with Seneca: "Innumerabiles esse, morbos miraris? Pharmacopolas numera!" "How truly grand and sublime," he continues, "is the aspect of Mesmerism, compared with the obscenity of medicine!" He next proceeds to lay the foundation of a magnetic pharmacology; and the medicament which he exclusively preaches up is, of course, the "physico-dynamic influence of one organism over another." Man can exercise, he says, this influence over other animals, and other animals over man. If you stare courageously at a wild horse, you tame him. The Tartar, who is always on horseback, becomes in time something of a horse himself. The shepherd's occupation renders his character sheepish; the swineherd assimilates himself to the swine he tends; the gosherd is as stupid as her geese; and the Greenlander, who lives on nothing but the flesh of the sea-dog, becomes in time a sort of sea-dog himself, "both in body and soul." To prove the curative influence of one organism over another, he asserts that a cat is an excellent remedy in typhus; that a rabbit (and no other animal but a rabbit) resolves indurations in the testicles, if it be laid upon them some hours a day; that turtle-doves (and turtle-doves only) are capable of flying away with the erysipelas, if left in contact with an erysipelatous patient. A vegetable organism, he continues, is also capable of exercising "a physico-dynamic" influence over an animal one; and such influence will be found far more powerful and efficacious than the operation of its cold remains and ashes, of whose medical virtues the foolish apothecary boasts. The following is the system of treatment which Dr. Riecke proposes for a phthisical patient: First regulate his diet, and the temperature and composition of the atmosphere he is to breathe; let him be generally magnetised twice a day, and let him sit for five minutes every two hours, in a baquet composed of two stems of digitalis, one henbane and three valerian plants, with perhaps the addition of a couple of rabbits; finally, let him wear, if circumstances require it, a magnetised woollen jacket, and a belt of zinc. The magnetic virtues of light, we are informed, are undeniable; and concentrated green light is proposed as an excellent application for ulcers. We cannot pursue the author in his discussion on the propriety of curing diseases by thunder and lightning, electricity, galvanism, mineral magnetism, sound,* odours, vapours, &c.; nor shall we at present attempt to enforce on the governors of our hospitals obedience to his injunction, that operative surgery be instantly taken out of the hands of the "imps of torture" (*Marterknechte*) who at present exercise it, and assigned to Mesmeric surgeons. The author winds up with a dream in which he realizes his system, (he theorizes only when wide awake,) and concludes by telling the reader, who should ask him when all this will be realized, that he does not know. But, he continues, "Everything has its time. When Peter preached at Jerusalem, 10,000 Jews were converted in a single day, but now there

* The hydrogenic harmonica, the jew's harp, the report of cannons, and the *tout ensemble* of an operatic orchestra are enumerated as specimens of sounding remedies, and the two former particularly recommended.

are 10,000 preachers, and not one Jew is converted. When Luther held forth, whole countries came over to him at once, but now a whole religious sect has the greatest difficulty to persuade a single individual to join it. I say to thee, Everything has its time, and Mesmerism will have it also, and that great time is approaching." Such rhapsodies as these only become worthy of our attention, as forming the first article of a periodical which is the organ of a numerous school, and which is edited by a professor of medicine, who has treated thousands of patients according to the principles which it embodies, whose magnetic experiments have been honoured by the presence of such men as Schleiermacher and Steffens, of whom we have never seen any condemnation by his colleagues or by the Prussian government, and whose magnetic cures we find duly registered in Hufeland's "Library of Medicine," amongst the other authenticated and important discoveries of the current year.

Mesmerism was not everywhere in Germany so faithfully and fantastically followed out and expounded as by the above professors. If some of its disciples saw in it a subject for the wildest, most impracticable, and incoherent speculations, others were not wanting who interpreted its phenomena in the grossest and most disgusting manner; and the materialism of these is as revolting as the flights we have been examining are ridiculous. The school to which we now allude is that of Gmelin, Kluge, and others, who appear to reject all the transcendental portion of Mesmer's philosophy, and seek for an explanation of all magnetic phenomena in the atmosphere of the nerves; for the demonstrable existence of which they appeal to the experiments and discoveries of Reil. This is not Mesmerism modified, but Mesmerism abolished; or, if such a change be regarded as part and parcel of the development of the science, then is its progress so Protean that, at each succeeding step, not a vestige is left of its former self. The fact is, we lose here all traces of the universal fluid, and of the "physico-dynamic influence" which had existed from all time, and had acted through all space; and we find installed in their stead a nervous atmosphere, upon which it is impossible to philosophize with any effect. Those of whom we now have to treat have deserted Mesmer in practice as well as in theory: they have given in to the fatal heresy of somnambulism; but this is not our present topic, which is the simple enquiry, not into what animal magnetism is, but into what its supporters say it is; and we shall find that they are not nearly so unanimous on the subject as is the public. When Gmelin, a plodding, plain-speaking German of the old school, is asked what animal magnetism is, he says, "The act of magnetising and the act of generation are essentially the same, and differ only in respect to the vehicle of communication." (" Die Begattung ist im Grunde nichts anders als thierischer Magnetismus, und unterscheidet sich nur durch das Vehikul.") If you remark, that "to vouch this is no proof," he is quickly ready with the latter, which he ingeniously finds in the fact that "Beischlaff" and "manipulation," (he terms magnetising "manipulating,") produce upon him precisely similar effects,—viz. weakness, indigestion, and general weariness. He states his evidence in language which we cannot venture to translate: "In der Nacht *cohabitirte* ich in der Absicht mich noch mehr zu schwächen; es geschahe mit Ergiessung," (p. 45:) he found himself the next day magnetically impotent! After manipulating, he does not feel the slightest inclination to sexual intercourse. His researches in the *science* are all, as may be supposed, of the most practical kind. Neglecting altogether those sublime enquiries into the nature of the fluid in which the universe is immersed, and rejecting unceremoniously all physico-dynamical theories, he institutes experiments to determine whether, if he manipulates a woman too vigorously, the perspiration which ensues is any barrier to the communication of the magnetic fluid. It is a *fact*, he solemnly assures us, that he could manipulate any woman but his wife. The reader may wish to be informed what Gmelin's particular manipulations were: we are ignorant; but, for the satisfaction of the scrupulous and delicate, he takes care to state that, whatever they were, they excited in him no lascivious ideas, "—viel weniger eine samenergiessung!" (p. 28.) All his writings show to what abuses the practice of this *science* of animal magnetism may give rise, concerning which the Baron Dupotet de Sennevoy exclaims, "If any person should ask what is the moral tendency of the doctrine of animal magnetism, I should answer that it obviously tends to establish the spiritual ascendancy of man over those material conditions which, in his ordinary state of being, fatally restrict the apprehensions, capacities, and comprehensions of the soul." The German would seem to come to a somewhat different conclusion, in an aphorism which he publishes without the slightest hesitation, and which is "that the bestial in man borders on the angelic." ("Das Thier im Menschen gränzt an den Engel." p. 206.) Such a writer as this is, in one sense, most precious: not that we could for an instant think of accusing *all* magnetisers of similarly revolting views; but that he plainly shows what this animal magnetism is easily capable of becoming, and justifies us in concluding that, if a blockhead, who evidently means no harm, proceeds to such extremes, there must exist amongst his brethren many "a closet lock-and-key of villanous secrets." We do not suppose, however, that Gmelin tells the world all he witnessed: "this honest creature, doubtless, sees and knows more, much more, than he unfolds;" but we must not quarrel with him on this score, for he confesses that he has seen one woman thrown by a magnetic process into a *furor uterinus*, (p. 119;) and that he himself magnetised away the modesty of at least a couple of girls. His notions on somnambulism we shall have occasion to mention when we come to discuss that spurious branch of Mesmerism: for the present we must leave him, merely observing that he declares upon his honour ("betheuert bei seiner Ehre") that, as a matter of taste, he would as soon "manipulate" an old woman of seventy as a girl of seventeen.

Kluge, the principal surgeon to the Prussian Medico-chirurgical Pepinière, in the well-written and laborious production of which the title is given at the head of this article, professes also, as we have stated above, the doctrine of a nervous atmosphere, which he appears to believe is capable of being bottled up in one subject, and then decanted into another, and which he considers as the agent of all magnetical operations. The character and situation of this writer preclude the possibility of his publicly indulging in such confessions or conceptions as those of Gmelin: accordingly, we hear nothing of the latter's very peculiar ideas, but he is repeatedly quoted as a respectable authority. Kluge, who, with the exception perhaps of Ziermann, is the least irrational of all the writers whom we have so far passed under review, is nevertheless as childishly credulous as Riecke is crazy and Gmelin disgusting. Such stories as the following, which is given as a proof of the existence of a nervous atmosphere, abound in his work: " A French nobleman of high rank (De la Tour Landri), during a visit to London, produced such a remarkable effect upon a young shoemaker, of whom he had ordered a pair of shoes, that the latter became senseless, fainted, and bled profusely at the nose, both when he took the measure and when he brought the shoes to be tried on. Surprised by the repetition of this scene, De la Tour made enquiries respecting his extraction, and found that he was born in France, but had been taken, in his childhood, first to Bohemia and then to Holland. De la Tour now recollected that the son of a sister of his, who had died in childbed, had been consigned, immediately on her decease, to a nurse, of whom and of her charge nothing had been heard since that time; he also remembered that the child was born with a remarkable mole between his shoulders. He instantly examined the young shoemaker, found that he bore the above mark, and convinced himself, after a few rigorous enquiries, that this person was no other than his nephew, the Baron de Vesins."

To prop up his theory of a nervous atmosphere, and of the effect of its mesmeric communication, Kluge actually relates again the old story of the girl married at Paris against her will, who dies or appears to die, is buried, and afterwards disinterred and resuscitated by her lover; and he claims all the merit of this resuscitation for magnetism.*

Kluge discusses at some length the relation of the ganglionic to the cerebral system, and the influence of the passions or will upon the operations of the nervous atmosphere. On this subject he mixes up some truth with a number of fantastic arguments and illustrations, citing instances of persons who could produce or suppress at pleasure, merely by exerting volition, morbid feelings, and phenomena. He is acquainted with men of a lively imagination and a firm will, who, by merely thinking on it intensely, can produce, in a few seconds, an erysipelatous inflammation in any part of the surface of the body they please. He is far fonder of stories of this kind, illustrating, as he thinks, the nature and functions of the nervous fluid, than of attempting to explain to us its *modus operandi;* and we are left quite in the dark as to how it drew blood from the nose of the Baron de Vesins; or how it revivified the Parisian girl; or how the will prevails upon it, as in the last-mentioned cases, to exhibit erysipelatous or other morbid phenomena.

Kluge wrote at Berlin in 1811: we leave that city, and arrive at Jena in 1822, and find that there, in the hands of Professor Kieser, Mesmerism has assumed another new face, and resembles no more Kluge's idea of the science than it does that of Mesmer, to whom indeed it is now merely indebted for its name. The third appearance of this strange essence is in the shape of a "telluric spirit;" and, in accordance with this new name, somnambulism is called "telluric life."

Kieser does not confine his magnetic phenomenon to the earth, although the "telluric spirit" plays the most prominent part in his sys-

* Wolfart has magnetically resuscitated a boy who had been fairly drowned, having been under water half an hour. (ΑΣΚΛΗΠΙΕΙΟΝ, p. 920.)

tem: he makes it out that we are magnetised by the moon every night, and unmagnetised by the sun every morning. Opinions somewhat similar to his are entertained by Nees von Esenbeck; and this school, to which we believe Kerner and Eschenmayer more or less belong, presents the latest phase of magnetism, and is considered to have raised the science to a very high degree of perfection. We cannot here treat at any length of the doctrines of these philosophers, which are much more complex and complete, much more harmoniously developed and carefully set forth, than any we have previously examined, and which enjoy the sanction of some of the highest names in Germany; but we shall shortly have some very edifying opportunities of knowing them by their fruit. In Kieser's scheme, we find nothing worth notice on the *modus operandi* of the 'telluric spirit;' of the action of which, again, we find no other result mentioned than somnambulism.

After having detailed the fate of Mesmer in his native country, where we find that he has been first assassinated, and then magnanimously or magnetically brought to life again by each of his successive friends, with the exception of one faithful band,—so that, were Kluge now to drag him from his grave at Frauenfeld, in Thurgovia, he would neither know "himself nor feel himself to be,"—let us next pursue the very different course which his doctrines took in France, where, in the national spirit of scepticism, we shall shortly find disciples denying their master, and mesmerising without the aid of any fluid, "universal," "nervous," or "telluric." Deslon, one of the earliest converts, and at first an intimate friend of Mesmer, was an ardent advocate of the "universal," and does not seem, on any material points, to have differed from his preceptor. Awkward circumstances, however, compelled him to stretch his theory now and then. Having undertaken to demonstrate to the commission, composed of Franklin, Lavoisier, &c., that an apricot-tree, magnetised by him in an orchard at Passy, near Paris, would throw one of his male patients into a crisis as soon as he touched it, it unluckily happened that the patient, who was introduced into the orchard with his eyes bandaged, and led from tree to tree, wanted discernment to distinguish the right one, and fell into a fit under one standing four-and-twenty feet from that which Deslon had "manipulated."* To explain this distressing mistake, Deslon had recourse to the idea which he seriously insisted on, that the apricot-tree had magnetised all the others; which explanation was doubtless suggested to him by the circumstance that the patient had manifested critical symptoms, of one kind or other, at every tree he came to. Such conclusions as these show that Deslon was ill calculated to administrate the new system in the absence of its author. M. de Jussieu, who formed one of the above-mentioned commission, was a partial convert to magnetism, and published, in 1784, a separate report in its favour; which, however, is so qualified, so opposed to all the essential points of Mesmerism, and based on such scanty evidence, that the *science* is more injured by one such advocate than by twenty violent adversaries. The following is the conclusion to which he arrives:

"The theory of magnetism cannot be admitted so long as it is not developed, and

* Bertrand, p. 113.

supported by solid proofs. The experiments made to establish the existence of a magnetic fluid prove only that one man produces upon another, by friction, contact, and in some rare cases by simple approximation (*rapprochement*), a sensible effect. This effect, attributed to a universal fluid, which has never been demonstrated, is evidently owing to the ANIMAL HEAT existing in the body, which emanates from it continually, is carried to a considerable distance, and may pass from one body into another. Animal heat is developed, augmented, and diminished by moral and physical causes: to judge by its effects, *it partakes of the properties of tonic remedies*, and produces, like them, results salutary or prejudicial, according to the quantity communicated, and to the circumstances under which it is employed. *A more extensive and discreet use of this agent will render us better able to decide on its real effect, and on the degree of its utility. . . . No person ought to be allowed to practise magnetism, except under condition that he promptly publish an account of the method of proceeding he adopts.*"

Magnetic tractors, vases charged with mesmerian fluid, or mirrors reflecting it and throwing it in all directions, in short, all its magic apparatus, Jussieu sacriligeously sweeps out of the temple of Isis. The votaries of health, too, who crowd around her shrine, he rudely pushes out of doors; telling the phthisical that they were better anywhere else, bidding the scrofulous and dropsical begone to the hospital, and giving the paralytic such cold comfort that they are fain to return to their miserable beds. The crises and convulsions, and indeed all the performances at the "tub,"—the cardinal points which constitute the soul of the system,—this sceptical believer decidedly condemns as extremely prejudicial, except in some very rare cases. To give a little original colour to his notions, he imagines that the animal heat may be the "electric fluid animalized," and disports himself in a lively description of its magnetic play. But all this is pure caprice; and, in fact, he only differs from his brother commissioners in the view he takes of four cases which they witnessed together at Deslon's; and these, even judging of them from his statement, were, as Bertrand observes, altogether inconclusive. Jussieu, however, has long been appealed to as an impartial witness to the truth of Mesmer's doctrines, although he denies them all, sets up a little scheme of his own, and can only cite four equivocal facts to support the latter, and justify him in dissenting from the opinions of the other commissioners.

One of the first revivers of animal magnetism in France after the revolution was Deleuze, who entertained the following notion of the nature of the magnetic fluid with which he operated; "I believe in an emanation from myself, because magnetic phenomena are produced without my touching the patient: *ex nihilo nihil.* I am ignorant of the nature of this emanation; I do not know whether it is material or spiritual, nor to what distance it can be made to extend; but this I know, that it is impelled (*lancée*) and directed by my will, for, when I cease to will, it ceases to act."

There is no case of individual delusion connected with the history of animal magnetism more painful to contemplate than that of M. Georget. Let us first hear what this writer has to say respecting the agency of the will in magnetical operations, as we shall shortly find that a fatal schism on this subject exists amongst the professors of the *science.* "It is necessary that the 'two parts' of the 'magnetic element' (he means the two individuals concerned in the operation,) should direct, as intently as possible, all the cerebral action towards the production of somnambulism; that both the magnetiser and the magnetised should will or desire that it be brought about. *I have found nothing more easy than to establish this fact.* Whenever I was '*distrait*,' my thoughts wandering to other things, or my mind ill at ease; whenever I did not direct my attention to the operation, I could often produce absolutely no effect." Here, then, we have two somnambulisers laying down the law,—and this law has the high sanction of no less a personage than the Baron Dupotet de Sennevoy,—that the will is an essential agent in the magnetic operation. The Baron is pleased further to inform us, "that the operation may be said to be almost purely intellectual; its success depending on the energy of the will."* But what says the plain-spoken Gmelin? He gives us very clearly to understand that the operation is not "almost purely intellectual;" and he states expressly that, as a magnetising agent, the will is powerless (der Wille ist kraftlos). Mesmer and his disciples never invoked the will, that we hear of, and got on very well without it; and, moreover, Bertrand, a somnambuliser, whose work was published in February, 1826, distinctly asserts that the influence of the will is a mere fiction, that he has somnambulised numbers of patients without its aid; and he further gives cases to prove its neutrality.† How are these contradictions to be explained? Dr. Deleuze produces an effect by his will exclusively, which Dr. Bertrand produces equally well, excluding his will: for effects to be precisely similar, we have always been taught that their causes should somewhat resemble each other; and here the only solution of the difficulty, which we can think of, will be found in the Latin law of one of the learned doctors, *ex nihilo nihil;* and it may very possibly be the true one. Leaving them, however, to settle between them this vital question, we return to M. Georget, who made the following declaration on his deathbed: "In my Physiology of the Nervous System I boldly professed materialism; but, scarcely was it given to the world, when new meditations on the extraordinary phenomenon, somnambulism, no longer permitted me to doubt of the existence in us, and without us, of an intelligent principle, altogether different from material existences—*let it be, if you will, the soul, or God.* I have, in regard to this, a profound conviction, founded on facts which I believe to be incontestible. This declaration will not see the light until there can be no longer any doubt of my sincerity, or any suspicion of my intentions." Now, were the evidence on which this conversion is founded complete, we should still be inclined to regard the latter with great suspicion, looking to the manner in which it is detailed, and to the source from which the evidence is drawn. The facts, one would anticipate, must have been essentially very equivocal on which such a loose creed as this was founded, (of which the vagueness strangely contrasts with the solemnity of its avowal.) Nor is this anticipation false, as we learn from the following extract from a speech of M. Velpeau, at the Academy of Medicine: "M. Georget became a zealous partisan of magnetism, after having been its opponent, and admitted its truth in his work on the Nervous System: he had performed experiments, and believed them incontrovertible. M. Londe assisted at these experiments. Well, Georget

* Dupotet, p. 151. † Bertrand, Preface, p. xxvii.

carried with him to the tomb his belief in magnetism; but M. Londe has outlived him, and you have heard him declare in this assembly that Georget and himself had been deceived, that they had been duped by some miserable creatures, who have since boasted of the circumstance."* What a satire is this on the manner in which pseudo-philosophers jump at the most serious conclusions! Here we have a "savant," whose only argument for the existence of his own soul and of a God, is derived from the tricks of a law student, who, having quarrelled with his friends, and lost all means of existence, feigned paralysis, and entered an hospital, where he soon became a noted performer of magnetic miracles! A magnetiser of high standing at Paris, Professor Rostan, entertains a theory of his art very similar to that of Kluge: he holds that the nervous fluid of the magnetiser, impelled by his will, may convey that will to the somnambulist; which is nearly tantamount to saying that a waggon carries the horses which draw it.

It is surely a sign of the worthlessness of a cause, when its advocates become less and less able plausibly to defend it; and that science must be radically rotten whose latest supporters are its worst. Now, this is the case with animal magnetism, which we confess has been propped by respectable names in its day, but which has now, in an evil hour, encountered the patronage of the Baron Dupotet de Sennevoy, whose officious assistance it cannot long survive. After a science has existed for two generations, we are justified in demanding, if not unanimity in its supporters, at any rate a definition from some one of them, and principally from the contemporary who has had the benefit of the experiments and arguments of all the rest. Nay, we are quite justified in denying the existence of a science of which there is no intelligible definition; for how can that proposition be even entertained, on the nature and identity of which it is impossible to obtain a distinct idea? We acknowledge that Mesmer was intelligible; that Wolfart's positions are perfectly comprehensible, absurd as they are; that the modern French school, too, lays down, for the most part, distinct premises; that accordingly, both in France and Germany, there are extant systems of animal magnetism, which one knows how to describe, and with which, consequently, we can practically deal. But, in whatever shape this unlucky doctrine may be met with abroad, it can assuredly lay little claim, in England, to either form or figure. Dupotet, in embracing it here, has squeezed the spirit out of it, and left its body without any marks or members by which to distinguish it. What are we to make of such expressions as the following? "Animal magnetism is that active principle which we possess within us, and which, under the energy of our volition, manifests itself by the effects it visibly induces."† "It is important to remember that the magnetic *power* does not consist in mere *gestures: another medium* is necessary, which the *manipulations* merely bring into play at the command of the will. This medium may be termed the vital principle, life spiritualized, universal, magnetic, or nervous fluid; *it matters not.* But, most assuredly, there is an emanation of a peculiar agent; for, *out of nothing, nothing comes.*"‡

* Lee on Animal Magnetism and Homœopathy, p. 44.

† Dupotet, p. 30. ‡ Dupotet, p. 151.

With respect to the emanation aforesaid, of a peculiar agent, we have now a remark or two to make. Georget, Deleuze, Rostan, and Dupotet make this and the will essential to the performance of the magnetic operation, which with them is nothing else, and can be nothing else, but the action of the latter upon the fluid in question; but the great discoverer of Mesmerian somnambulism, Puysegur, not only attached very little importance to manipulations, but never had recourse to the theory of a nervous fluid; whilst Bertrand denies that either the latter or the will have anything to do with the production of somnambulism. Barberin somnambulised his patients by merely praying by their bedsides; and the Abbé Faria produced the phenomenon in question by commanding those whom he treated, in a loud and imperative tone, "to sleep."* Do not all these contradictions, both in theory and practice, plainly show that all the so-called magnetic phenomena originate exclusively in the imagination?

The doctrine of a magnetic fluid, however, is so vital a part of the present system of Mesmerism, that it is worth while to examine it a little more closely. Alleged proofs of its existence may be divided into three classes; viz. those derived from the cases of somnambulists who have seen it; those deduced from its isolation in metals, &c.; and those drawn from its pretended effects where the somnambulist was not conscious of the presence of her magnet. Now, with respect to the first class, Bertrand very properly observes, that a somnambulist will see anything, and that there is nothing she cannot be made to see. The lady always shows her gratitude to her magnetiser, by bringing him intelligence from somewhere or other, often from the other world, in confirmation of his theory. Where the operator deals in a nervous fluid, the patient sees it streaming upon her from the ends of her fingers: where he thinks proper to deny the existence of a fluid, she can discern nothing of the sort, and describes the somnambulic trance as being brought about by *very different means.*† Women, magnetised by disciples of Swedenborg, preached the doctrines, both metaphysical and religious, of the latter, and had visions confirmatory of his views. The peasant, magnetised by Puysegur, appears to have altogether lost his identity, and to have become part and parcel of the being of the latter. The Prophetess of Prevorst, who had a mystic tellurist for her physician, had interviews and long conversations with spirits, and talked very learnedly on her solar and vital circles, which have been only explained by Görres according to the principles of modern German philosophy. Finally, a religious lady at Weimar, who magnetised her maid-servant, was able, through the latter, to communicate with God himself, who used to appear to the girl, and talk to her in a very familiar manner.‡ Now, knowing all this, we are surely justified in denying admission to the evidence of somnambulists, who shut their eyes to see what they could not see with them open. With respect to the powers of magnetised water, magnetised handkerchiefs, magnetised sovereigns, &c., we doubt not their miraculous efficacy; but we cannot allow that magnetism has anything to do with it. Bertrand sent a

* Bertrand, p. 246.
† See Bertrand, p. xi.; who does not, however, inform us what these means are.
‡ See the work, Fingerzeige Gottes, &c.—Weimar, 1838.

magnetised handkerchief to a patient a hundred leagues off, and it somnambulised her instantly; nor could he at all account for this until he found that a non-magnetised handkerchief produced precisely similar effects. Mesmerised water produces most astounding phenomena, but common water is capable of causing precisely the same; and the former cannot at all compete with that drawn from the well dug near the tomb of the deacon Paris, in the cemetery of St. Medard, in the French capital, which one of the devotees of the said deacon could easily distinguish from all other water; although his brother, a decided adversary of Paris and his fanatical sect, tried to deceive him. Bertrand states that he instituted experiments on the subject, but was never able to produce any effect on a patient, when awake, by touching her, *unknown to her*, with magnetised objects. This writer formerly believed in the existence of a magnetic fluid, but changed his opinion in consequence of his own researches. He was a somnambuliser, who seems to have had less excuse for practising the art than its more ardent professors; for he does not appear to have anticipated any benefit from it, and he describes the painfully degrading scenes to which it frequently gives rise with the greatest *sang froid*.

The third class of proofs which we have to consider is that deduced from the direct influence which the magnetiser is said to be able to exercise over his patient, when he is absent from her. On this subject, Bertrand says that the utmost precaution is necessary, "when experiments are made respecting the production of somnambulism on persons who are adapted to this state, as it were, (*pour ainsi dire façonnés à cet état*,) by daily experience. It cannot be too often repeated, that a gesture or a glance of the magnetiser, *nay, even the mere thought occurring to them that he wishes to act upon them*, suffice to produce somnambulic phenomena."* He then cites a case in confirmation of these assertions, which is so conclusive, conveys such a perfect idea of the manner in which magnetic experiments are conducted, and throws such a strong light on the source of magnetic phenomena, that we shall translate it, with some abbreviation; convinced that one such statement made by a somnambuliser, in perfect simplicity and good faith, will save us the trouble of commenting on the crowd of "facts" of a similar nature, which have been published as the foundation of new and marvellous truths.

"I studied," says he, "during some time, the case of a somnambulist, Madame Chevalier, whom I did not magnetise myself, and on whom the *lady* who treated her exercised an influence which was really extraordinary. She caused, for instance, at will, the paralysis of an arm or of a leg, or simply of a hand or finger; she could deprive her also of speech, hearing, and smell. But her power was not limited to a local action; *she could paralyze, so to say, at a blow, all the parts of the body of the somnambulist, and throw her into a state of complete and general insensibility and immobility, which constituted a veritable lethargy*. In order that I might better be able to estimate the value of these marvellous appearances, Madame D. *was kind enough to enable me to reproduce them at will*, by putting me *en rapport* with her patient. However, my first care was to ascertain what share the imagination had in the production of these remarkable phenomena. It was customary, when it was wished to throw the patient into a lethargy, for Madame D. to signify her will by passing her

* Bertrand, p. 269.

hand quickly before her, from above downwards. After having several times made this *passe,* with a concomitant exercise of the will, and always successfully, I made it without exercising the will, and I succeeded just as well. I now tried the power of the will without the *passe,* but with no effect. Then, in order to ascertain whether the *passe* and the action of the will together would affect the patient, unknown to herself, I performed the usual operation, separated from her, first by a wall, and afterwards by a simple partition or a door; but always in vain. . . . The real cause of the lethargic phenomena was shortly revealed to us. Madame D., being one day forced to absent herself on business, *left her patient to be unsomnambulised by some magnetisers who were in the room.* I was there also, magnetising another woman, when suddenly Madame C. ceased to answer the questions which were addressed to her, and fell into the lethargy which it was usual to produce in her: it was immediately found that she was insensible. *This being clearly established,* I wished to see whether the other somnambulist *could give us any information in the manner in which it had been produced*; and I said to her, 'Look at Madame C.; tell me that which is taking place within her, and why she has fallen into her present state.' The women directed her attention as I had ordered, and, instead of answering me, fell herself into a *state of insensibility, and appeared, in short, dead.* I was not able to restore her for some minutes.* At length she was able to speak to me, and she said with a laugh, which was habitual to her, (*her intellectual faculties being in a state bordering on idiotism,*) 'Ah! you're not up to it; you'll have some terrible trouble. She's paralysed. Madame D. is only gone out to act upon her at a distance; and, if she doesn't return, you'll not be able to get her out of the state she's in.' I thought at first that this really might be the case; but time passed on, and Madame D. did not arrive, nor could she be found in the neighbourhood. She had been seen to leave the house, and go towards the place where she said she had business. I now began to be seriously uneasy. The somnambulist remained in precisely the same state, that is, *dead to all appearance;* and I could not but anticipate that the most serious evils would result from her continuing in this condition. She herself had said, whilst somnambulised, that, *if left in lethargy for more than ten minutes, she was in danger of permanent paralysis of the extremities, and of at once losing her life.* I shall not try to paint the *anguish* (the expression is not exaggerated) which I now endured. At length, by great attention, and after using *efforts of every kind,* I succeeded in restoring the patient to the state of somnambulism, *without her suffering any other evil results than a violent pain in the head, and a sort of 'étourdissement,' for which she ordered herself a strong dose of magnetism, which was administered to her.* She assigned the same cause for her fit which the other somnambulist had done. Mad. D., she said, had magnetised her at a distance, in order to make her fall into paralysis; but her action had not been sufficiently powerful to cause it to cease. The next morning I learnt, though without astonishment, from Madame D , that she had never thought of magnetising her patient whilst absent, and *that therefore I had been indebted to the imagination of the latter for the scene which had frightened me so much. She had thought that this was an experiment,* and that the persons left in the room were there to divert her attention, and render it more conclusive. In giving this case in detail, I have had for my object, not only to prove the power of the imagination, *but to familiarize a little my readers with the singular scenes which may daily occur in magnetic treatments accompanied by somnambulism.*" (*Bertrand,* p. 270.)

One of the facts most conclusive in favour of magnetism at a distance, and of the existence of a magnetic fluid, is the following experiment tried on Mlle. Samson, at the Hôtel Dieu: "a person who had for fifteen days previously been the subject of '*une multitude d'expériences,*' of which a great number had been instituted, for the purpose of exercising some influence over her, unknown to herself." M. Husson asks Mlle.

* It is very common to see somnambulists experience all the morbid symptoms of the persons to whom their attention is directed, *especially if they know what their symptoms are.—Bertrand.*

Samson if she is asleep? The latter answers, "that she is not sleepy, and that she never goes to sleep so early." She coughs, and M. Husson retires to a place where she could not see him, but where he also could not see anything that took place. The magnetiser begins to direct his action (*diriger son action*) at seven o'clock; at eight minutes past seven, the patient says aloud, talking to herself, "It's astonishing how my eyes hurt me; I can't keep them open." A minute afterwards she was asleep.* Bertrand was present, with several other orthodox somnambulisers, at this conclusive experiment, and with them authenticates the "fact," which occupies a prominent place in M. Husson's report; but, notwithstanding the deference he owes to the general opinion of his brethren, he feels compelled to observe, that Husson's question was calculated to make the patient suspect that she was about to be the subject of an experiment; and that her readiness in stating that she could not be sleepy, and her talking aloud to herself, show that she *did* suspect it.† All this is quite plain; and we very much fear that the magnetisers must soon acknowledge, strongly as they at present insist on its impossibility, that their vaunted phenomena, instead of having a real and sensible fluid for their instrument, are indeed *ex nihilo nihil,*—"distempers of the brain, begotten by a sickly fancy." If we have a magnetic fluid continually floating throughout our frames, and streaming from us on frequent occasions, the world surely would not have remained insensible to its operations for six thousand years. If there were, indeed, such "music in this little organ;" if man were, in very truth, "easier to be played on than a pipe;" we may be certain that the discovery would not so long have been deferred. The vulgar would have made it, ere the scientific could have dreamt of it; and our common life—not the hall of the somnambuliser—would be the field on which to contemplate its results.‡

If we allow that there is "an emanation of a peculiar agent," without which Dupotet confesses that magnetism is an absurdity, we are bound to enquire how far its operation extends; or, in other words, how far the animal magnet will carry. Somnambulisers in France and England have more exercised themselves in propelling their fluid through doors and brick walls than in projecting it to any distance. Not so in Germany, where the practised Jäger, brushing away the dew at early dawn,—ardent and yet an artist in pursuit,—afraid to shatter his som-

* Expériences de l'Hôtel Dieu, quoted by Bertrand, p. 263.

† We may here remark, that Bertrand is praised by Husson, and that he delivered public courses of lectures on Somnambulism in 1819, 1820, and 1821. Dupotet states that the objections of Bertrand to the value of the experiment on Samson were practically refuted; but we find no allusion to this in Bertrand, who is since dead.

‡ In Germany, however, where philosophy boldly anticipates all practical objections, our common life is beginning to be made sensible of the debt it owes to magnetism. Stahel, of Wurzburg, has lately published three works (which we have not yet seen) of Professor Hensler; one of which is "On the Influence of Animal Magnetism on the Health and Longevity of Man *in social life, and especially in the state of Marriage;*" another is entitled "Effects of Animal Magnetism on Man and Nature, and on its importance in a medical, juridical, philosophical, religious, and historical point of view, and in respect to *social community;*" the third is "On the *different Kinds* of Animal Magnetism, and on their *different Effects*, &c." The work on Magnetism and Marriage, the publisher promises himself, in his advertisement, will produce a great sensation in the public.

nambulist by too close a shot, brings her tumbling down at the distance of six good English miles. Though each to the other invisible, and each separated from the other by many a line of undulating hills, he aims at her* "between the eyes and breast," and she falls as perpendicularly as the racoon descends when invoked to do so by a Kentucky rifle. Nadler† shot at a woman at the distance of eighteen miles, and hit her precisely at the moment‡ at which he fired: no sooner was his will at the trigger than down she came. This patient begged her magnetiser not to try such experiments with her, as otherwise he might somnambulise her *when she was in a very inconvenient situation.* His love of science, however, was not to be damped by such scruples; and so he persisted, and was always as successful as at first: probably he anticipated that, were her fears realized, the *science* might be enriched with some very new and singular "facts." A married woman, whose magnificent "*soirées*" are described, and whose case is given at great length in Wolfart's Annals,§ could not hear her child cry, which was by the side of her, but could *feel* her magnetiser whenever he chose to make her, although he had left Berlin definitively, and gone (we presume) to Mecklenburg, his native country, there to remain. This lady was accustomed to choose her magnetiser out of the crowd of young medical men who came to visit her, and, whilst she was under treatment by one, she indicated who was to be his successor, *before she ever saw him.* Wolfart was her magnetiser in ordinary, and solemnized her successive unions with his scholars. The evening before Dr. Barez, the Mecklenburgh physician, ran away from her, (but not, as we have seen, with the intention of ceasing all communication with her,) she made an offer to a certain Dr. Oppert, to whom she had never spoken, and whose own literal account of the courtship is as follows: "It was merely on account of my having, when visiting, one evening, the patients of Professor Wolfart, several times directed my attention to her, *that I seem to have come into contact with her.*" In making him the offer, she was pleased to express herself to the effect "that she should be useful to him in assisting him to an improved 're-cognition' of magnetic operations." The reason she assigned for not remaining in magnetic widowhood was, that she did not like solitary somnambulism; she had an objection "to sleep" alone. Her offer was accepted, and the wedding duly solemnized. We copy the bridegroom's account of it: "On the 25th of March, at ten o'clock in the morning, I was introduced to her (for the first time) by Professor Wolfart. He magnetised her simply: she fell asleep, as usual, and then rose to the state of '*clairvoyance.*' Whilst she was in this, I was first brought *en rapport* with her, and the professor left us after a few trifling remarks." Now follows an account of the bridal dalliance. The first question

* "In a solitary place, at five minutes after five, I began to perform the process mentally; it lasted twenty minutes;—I took my course from her eyes to her breast. On arriving at her house, I heard that shortly after five she had fallen, complaining of pains about her eyes, had been convulsed slightly, and had shortly *waked* into a magnetic sleep."—*Weinholt* quoted in the ΑΣΚΛΗΠΙΕΙΟΝ, p. 890.

‡ "Ganz genau in derselben Zeit." He had regulated his watch by her clock, for the purpose of ascertaining the rapidity with which he could reach her, and fired, we presume, watch in hand.

§ Vol. ii. part ii. p. 88.

which Dr. O. put to her was, "How do you do?" which were also, be it remembered, the first words he ever addressed to her. She answered, "Very well, thank you; only my eyes hurt me." He then practised some manipulations, and enquired "whether he relieved her." "Oh! yes," she said, "you do now." "Do you remember the medicine you ordered for yourself on Friday?" "Oh! yes; the *pulmonaria.*" "What did you prescribe for yourself yesterday?" "Lay your hand on my head, and I'll tell you." The doctor did as he was told, and she answered "*Leontodon taraxacum.* But that is not the right; it only seemed to me so. This evening I shall be able to tell the professor what the other plants are —there are three more. He must rub my organ of place and my organ of colour." In such a frivolous and foolish tone as this is the conversation continued through several pages.

Having presented our readers with a tolerably complete view of the various theories of the magnetic agent, we next turn our attention to its pretended effects on the human system, adopting the descriptions and details of magnetisers themselves; and on the very threshold of this subject we encounter an important contradiction, which is, that Mesmer magnetised for years without ever inducing somnambulism, whilst his present followers mesmerise without ever scarcely producing anything else; though none of them has dared to assert that their process differs essentially from that of the founder of their sect. Should they now, however, hazard this explanation, we have only to appeal for its utter refutation to Dr. Andresse, of Berlin, who, though he practises precisely according to the modern formula, assures us that, of a great number of patients whom he has treated magnetically, he has never thrown one into somnambulism.* The school of Wolfart regards this phenomenon as an insignificant accident in the course of Mesmerian treatment: nay, the professor goes so far as expressly to declare *that it does not belong to magnetism, and that the latter presents the means of combating and avoiding it.*† He holds that it cannot occur in healthy subjects, and that the liability to it arises from disease of some of the viscera. Mesmer's anathema against it we have already had occasion to mention. The fact is, the woman magnetised is more or less wittingly the child of her own fitful fancy,—γυνη γαρ ουδεν οιδε πλην ο βουλεται;—her fancy is moulded to the will of her magnetiser, and, whatever symptoms the latter chooses to summon forth are sure to come, whether they be somnambulic, paralytic, or critical. If he could view the cloud of facts which in our day threaten to obscure the sun of reason, the Greek philosopher would only repeat his old exclamation, Ως ες' απιςον η γυναικειη φυσις. Wolfart states that magnetism may cure a patient, without producing upon her any sensible effects; but that this is not frequently the case. He divides the phenomena generally induced by Mesmerian manipulations into five classes, viz. 1. *Sensations agreeable, or the contrary:* the former where the disease yields at once; the latter where it can only be subdued by a "crisis." 2. *Alteration of temperature.* 3. *Convulsions.* 4. *Secretions and excretions of all kinds.* 5. *Changes of tone in the vi-*

* Wolfart's Annals, vol. i. part i. p. 168.
† For this startling assertion of the Berlin professor, see his Annals, vol. v. part ii. p. 187.

*tality of the senses and of the brain,* amongst which somnambulism may sometimes occur.* This system, it will be instantly perceived, is identical with that of Mesmer: with respect, therefore, to the reality and value of the above effects, we may refer the reader to the Report of Bailly, in which it is distinctly proved, as most of the somnambulisers of the present day allow, that they have their origin solely in the imagination, to which all kinds of stimulants were administered, both at Paris and Berlin.

Magnetic somnambulism is the great modern phase of Mesmerism, and respecting its production we find the usual mass of contradictory evidence. Puysegur, its inventor, used to produce it, amongst other simple methods, by merely wishing to produce it; Barberin made his patients somnambulic by praying; Faria, by shouting; and the plan at present in vogue is "pawing." Respecting the nature of this state, the opinions of the learned are equally divided. Puysegur affirms that it exalts the intellect to a superhuman pitch. He derives, he tells us, from a somnambulic peasant, who is more than half idiotic when awake, both knowledge and judgment. He denominates him his intelligence, and then describes him as a being for whom it is impossible to find a name. "When he is in a *crisis*," he continues, "I know no one more profound, more prudent, and more *clairvoyant* than he is." Health M. de Puysegur dispensed as liberally and as easily as did M. de Lauterbourg at Hammersmith terrace, five years afterwards. "I have only one regret," says he, "and it is that I cannot touch all who come." Unfortunately, the ideas and statements of this enthusiast are utterly at variance with the theories and observations of contemporary and subsequent German philosophers. Gmelin, who somnambulised at Heilbronn at the very time that Puysegur was hard at work at Busancy, sending the whole country to sleep, tells us that the intellect escaped its operation, and that the revelations made by the women he "manipulated" were of such a nature that he pitied equally those who made them and those who staid to listen to them. Moreover, Kieser, and the other more systematic and philosophic writers who see in somnambulism a prominent phase of ganglionic life, hold that the somnambulist necessarily descends in the scale of being, and assert that she is incapable of even *exercising* her intellect. They make of her one huge organ of perception, unable to perform a reasoning process; holding that her brain is paralyzed, and her whole vitality absorbed by the ganglionic system. They turn man into a mere animal by the very process which Puysegur, Redern, and Dupotet adopt to make him into a god. Where their hands keep in abeyance the higher attributes of humanity, the "paws" of Sennevoy "lead us to entertain the spirit of a philosophy which is of the most cheering description, annihilating all those dark attributes of materialism which have so long thrown a gloom over the paths of science."† What Wolfart considers a disease, Gmelin a sensual orgasm, and Bertrand a fit of idiotic ecstacy, the Baron recommends us as the means of moral and physical regeneration.

The descriptions of the phenomena observed in the somnambulic state

* Annals, vol. iv. part ii. p. 22.

† Dupotet, p. 345.

are as various as the characters of their authors: we shall cite here that of M. Husson, which has the merit of being simple and succinct.

"The patient who falls into somnambulism acquires a prodigious extension of the faculty of sensation. Several of his external organs, generally those of sight and of hearing, become dull, and all their functions are performed internally. The somnambulist uses neither eyes nor ears, and still he sees and hears better than if he were awake. He only sees and hears those with whom he is *en rapport.* He only sees that on which he gazes, and generally he only looks at objects to which his attention is directed. He is subservient to the will of his magnetiser in respect to everything which is not prejudicial to him, and which does not run counter to his ideas of justice and truth. He feels the will of his magnetiser. He sees, or rather he feels, the interior of his own body and of that of others; but he only remarks, for the most part, those parts which are not in the natural state, and which disturb the general harmony. He perceives the magnetic fluid. He recollects things which he had forgotten when awake. He has '*previsions*' and '*presensations*,' which may be often erroneous, and which are limited in their extent. He talks with surprising fluency. He is not exempt from vanity. He improves, of himself, during a certain time, if he is managed properly; he goes astray, if badly directed. When he awakes, he has not the slightest recollection of the ideas and sensations he has had during the state of somnambulism."*

Puysegur speaks thus of his somnambulic patient:

"Man in the magnetic state is to be considered as the most interesting being in existence. With regard to his magnetiser, it is through his unbounded confidence in you that you have been enabled to bring him completely under your control. It is, therefore, for no other purpose but that of benefiting him that you have any right to exert your power. Attempting to deceive him or abuse his confidence, while in this state, is to commit a dishonest action, having a tendency contrary to his benefit; whence it follows that a contrary result is produced to that originally contemplated." (*Dupotet*, p. 144.)

A Mr. Wright, magnetised by Dupotet, says that "the word *fascination* aptly describes the influence which the magnetiser exerts. With me it is not an intellectual fascination, but only physical and moral."† A patient (one of Dupotet's, we presume,) her life being in the hands of her somnambuliser, petitioned him to *murder* her. "Why do you call me back to life?" said she, in her magnetic exaltation; "if you would only go away, this body which oppresses me would grow cold, and my soul would no longer be here on your return. I should then be perfectly happy."‡ Dupotet thus comments on this interesting case: "All hold nearly the same language, and suggest the idea of the soul being partially disencumbered of the coils of its mortality." This, as we shall afterwards find, is almost precisely the view of Mr. Mayo. Bertrand states that "moral inertia" constitutes the most remarkable psychological phenomenon of somnambulism;§ that it explains the want of caution with which uneducated somnambulists answer all the questions which are put to them; and that it is liable to be abused in many cases.|| Ziermann dwells on the danger which the somnambulic patient incurs when the magnetiser quits her. "His absence," he says, "disturbs her, and causes her anxiety, convulsions, spasms, and fainting fits; all which symptoms are exasperated to the highest pitch if any other person than

* Quoted by Bertrand, p. 292.

† Dupotet, p. 56.

‡ Ibid. p. 166.

§ Bertrand, p. 292.

|| Ibid. p. 426.

her magnetiser attempt to offer her assistance; and even he cannot often allay them without great trouble and exertion. Her spirit frequently seems to be blended (*wie verschmolzen*) with his; and with him, consequently, her soul appears to depart and return." He advises his readers, unless they have plenty of time to spare, to enter into no very intimate "*rapport*" with a female patient; at any rate, into none "which is more intimate than natural;" or they may have great difficulty in getting out of it.* In another place he compares his somnambulists to his children, and gives rules for training them: some, he says, require the curb, some the spur.† Many, he continues, can frequently see nothing unless the magnetiser concentrate and direct their attention. We must go very rationally and orderly to work, he adds, or we shall turn out cases of somnambulism which will be a disgrace to ourselves and a scandal to the *science.* When the magnetic treatment has been delayed beyond the accustomed time, the most dangerous results, according to this author, may be expected to occur, especially in excitable cases. "Restlessness, care, and anxiety then trouble the patient; he misses the most important part of his life; he breathes with difficulty and embarrassment; he trembles, staggers, and faints. His face is as pale as death. The vital functions are arrested; the muscles relaxed; the blood is hardly felt to move in the veins; and the extremities grow cold. But, as if dispersed by the wand of the magician, all these symptoms fly at the approach of the magnetiser, who has but to touch that which was but just now to all appearance a corpse, and it starts into vigorous life."‡

Individual cases of a very suspicious relation between man and woman are not at all rarely met with, even in the works of magnetists themselves. Their very phraseology is equivocal; they have a habit of talking of each other's somnambulists, as if they were their wives or mistresses. Most magnetisers, it would seem, are initiated into the secrets of their science by some one lady, with whom they get into a very intimate "*rapport,*" who is known to all the world as their "crack" patient, and whose case furnishes the occasion of their magnetic *debut.* Kerner was converted to magnetism by the Prophetess of Prevorst, a Mrs. Hauffe, who spent the latter part of her life in his house. Wolfart served his apprenticeship to a lady of Berlin, suffering under *intumescentia uteri*, whose case fills whole numbers of the *Ασκληπιειον.* A magnetiser, mentioned by Dr. Andresse, of Berlin, was so closely *en rapport* with one of his patients, that she could scarcely be without him for an instant, ("sie konnte fast keinen Augenblick ohne ihn sein;") and this lasted for two years.§ Some magnetisers were introduced into the house of Prof. Flörke at Berlin, and one of them, a Mr. H., soon got possession of his daughter. Her parent (who is not at all afflicted, but very scientific,) thus describes the "fascination" of the latter: "She could bear nothing but what Mr. H. had magnetised. Even her most intimate friends she could not suffer to be near her, if they were not conducted to her by Mr. H., or brought *en rapport* with her by means of a magnetised flower which they held in their hands."|| Two years before Madame de P., whose case is related in the second volume of Wolfart's Annals, saw the

* Wolfart's Annals, vol. v. part ii. p. 17. † Ibid. p. 36. ‡ Ibid. p. 41.

§ Ibid. vol. i. part i. p. 173. || Ibid. p. 215.

latter, she had a vision in which he stood bodily ("leibhaftig") before her, and magnetised her. When she was actually under his care at Berlin, the most affectionate and unreserved familiarity existed between them: she made him the confidant of all her domestic secrets; and, on leaving Berlin to join her husband, she prophesied to him *that she should very shortly become pregnant by the latter;* which prophecy was duly communicated to the husband by Wolfart, who shortly afterwards published a history of the affair in his own Journal. The verses written and the revelations made by this lady during her course of treatment are of the most incoherent description; but they are all communicated to the public as authentic and highly valuable "facts." She writes letters to her husband whilst somnambulised, which she dare not read when awake, which betray an intimate acquaintance with the locality of Cleve, where he is residing, although she has never been there, and in one of which she says, "Dein Instrument hat mich oft zu dir herüber gezaubert," ("Thine instrument has often magically transported me to thy side,") although she had no possible evident means of knowing that one of his neighbours had lately lent him a piano. The husband is particularly edified by all this; he regards his wife as a superhuman being, and Wolfart as a wonderful creature. She addresses Wolfart with the singular pronoun "thou." One of her daughters, she tells him, has suddenly been seized with convulsions, and to cure her she goes on to say, "I thought on my friend, who is so kind to every one, and with thy will and thy sympathy, and knowing what thinking on thee did for me in the hour of danger, I breathed into her hands and magnetised her, as thou didst me when I was asleep. I feel myself for ever allied to thee, ('auf immer Dir verwandt,') but thy power and dignity I am too weak ever to aspire to." She then gives him particular cautions respecting his health, especially touching every feeling *in the left side of his breast;* recommends him magnetised herbs, and finishes with a burst of sublimity which is too unintelligible for translation. Wolfart, on the other hand, furnishes her with a regular supply of magnetised wool, plants, bottles, &c.; and also communicates with her in the distance, after the manner of Nadler and Wienholt; and by these means was always able soon to harmonize her system, however violently disturbed. On the 29th of March, 1819, Monsieur de P. thus writes to Wolfart: "On the 26th of this month, my wife was prematurely brought to bed of twins. She is as well as can be expected, and is much indebted to you for a magnetised bottle which you gave her two years and a half ago, which magnetised her after her confinement, and cured her of a terrible attack of spasmodic colic."

Of such stories as the following we have heard abundance in Germany, related by members of families in which the circumstances are said to have occurred. One day, whilst at Berlin, Madame de P., in a somnambulic trance, announced that a letter would shortly arrive from her husband. Suddenly she exclaimed "The postman is now coming down Frederick street." Dr. Ebel, one of the magnetic staff, who was with her, instantly walked from her couch to the window, and saw the postman coming down the street towards the house, which was at the corner. Before he reached it, however, he turned to the left down Behren street, and in the same moment the somnambulist said, in a tone of vexation,

"Ah! he's going round the corner: he's gone; but he'll be here directly." He came as she had foretold, and brought a letter from Cleve, 420 miles distant, which Dr. C. laid sealed upon the pit of her stomach, and which she then perused, detailing its contents to him. On waking shortly afterwards, she did not know that a letter had arrived. Amongst her somnambulic rhapsodies we find the following words: "How limited are the contemplations even of the most *clairvoyant!* Why and wherefore? Ask me not; for, by the eternal Godhead, I *dare* not say it." It is pretended that a mysterious change was operated in the whole character of this woman by somnambulism; that it invested her with a truly religious solemnity, and filled her with a spiritual peace which never afterwards deserted her; that, in short, "it stamped, as it were, another seal on her personality: ("Drückte gleichsam ein andres Siegel ihrer Persönlichkeit auf.")

The absolute immorality of somnambulising, and the abuses to which the practice is liable, are never expressly denied by French writers on the subject, who, if they can establish what they are pleased to call a "fact," trouble themselves very little about its moral bearings. In Germany, however, it has occurred to some of the more conscientious friends of the *science,* that objections on the score of common decency might be made to some of its practical results, and that they have accordingly undertaken to meet them. There is nothing like assuming a bold front in a bad cause; and we find this verified in Kluge's eulogy on magnetism, which he makes when called upon for its apology. To have remained on the defensive in such a weak position would have been fatal policy; and so he at once anticipates all attacks and objections, by gravely assuring us that, if a woman was not virtuous before, she will become so when somnambulised. He divides the magnetic state into six stages, in which, the more advanced the patient is, the more moral does she grow. In the sixth stage, says he, the patient is capable of no impurity; and, were he even a degraded being, he attains here to a sense of virtue. ("Im sechsten Grade ist mann keiner Unlauterkeit fähig, und selbst der schon Gesunkene erlangt in ihm zum Tugendgefühl." p. 238.) These interesting assertions are duly confirmed by experiments. A patient, whom her magnetiser once attempted to kiss whilst in a trance, went, we are informed, into convulsions, and soon afterwards died. Another, we are told, would not allow her physician to kiss her hand when asleep, though she would when awake. It would seem that all Kluge's female patients agreed to be virtuous for no other reason than that of preserving intact the reputation of the *science,* of which they were evidently much more chary than of their own. This author gives an account of a woman who, from the mere consciousness that a man was regarding her with lustful eyes, was afflicted with the most painful symptoms, which were only relieved by his departure. Though these patients were morally impregnable, their bodies were liable to be invaded by all the morbid symptoms of any person with whom they were placed in contact: two afflicted with different complaints have been known to change them for a time, and then each to return her own to the other! They had an innocent predilection,* it is true, for their own somnambuliser,

* "Eine unschuldige Zuneigung."

and for all that was his; they were exquisitely sensible to all his variations of temper and conduct; but they would not tolerate even the slightest liberty.*

The conclusions to which any unbiassed person would come respecting animal magnetism, taking all the details of its professors as authentic, are that, though furnishing means for every kind of immorality, it still promotes every virtue; that it performs all imaginable miracles, and cures all existing diseases, and at the same time creates a few more.

There are two classes of marvellous phenomena presented by somnambulism, one of which has reference to this world only, and the other to both this and the next. We shall treat of the former first. It is not the object of this article to undertake a formal criticism of these phenomena: we prefer writing the history of magnetism, and relying for our information on the unexceptionable authority of its ardent advocates, to appealing to its constituted adversaries and rancorous foes. The Reports of the French Commissions have already been often placed before the English public. With respect, however, to the value of the evidence on which the reality of such an accomplishment as that of seeing with the eyes bandaged is based, we will just observe, that arguments drawn from the respectability of the persons who attest it we cannot admit; inasmuch as we shall find that persons just as respectable have formally attested the greatest absurdities that were ever published. It is one of the peculiarities of animal magnetism that its grossest outrages on common sense will be found to be those of its facts which are best authenticated. We may observe that Bertrand, who somnambulised for years, admits that he never witnessed a fact which convinced him of the existence of this phenomenon. Finally, we would advert to the evidence furnished by the examination of M. Berna's somnambulist by Bouillaud, Roux, &c. in 1837.† Here we find a girl who has reason to suppose that a plain card, which was held up to her when her eyes were bandaged, was a playing card, stating that it was the knave of clubs. We hold this failure to be much more conclusive and instructive than any of the attested facts on which magnetism is built: if she had merely expressed her inability to state what the card was, we grant that then the experiment would be about as valueless as most of the positive evidence in favour of magnetism; but, as it was, she at once betrayed her own character, that of her magnetiser, and the nature of the whole business: either she had an impression that the card was the knave of clubs, and so was deceived herself, or she merely sought to deceive others, and was, in that case, a deliberate impostor.

Kluge states that such is the sympathy between the magnetiser and his somnambulist, that he has known the latter to vomit and be purged in consequence of medicine which the former had taken. He once put his watch to the ear of one of his somnambulists, and she could not hear it tick; but she heard it in her left ear as soon as he put it to his right. Whenever he put pepper on his tongue or drank wine, she tasted these things distinctly on her palate. Indeed, he warrants the conclusion that his somnambulists were extensions of his own being, appendages to his

* Yet the man who asserts this adds, "Auch Gemüthsaffekte des Magnetiseurs können während der magnetische Behandlung auf die Kranke übergehen." (p. 202.)

† See Lee on Animal Magnetism, &c. p. 37.

own body, which, though he did not feel them, were as sensible to his pains and pleasures as his other limbs; but which, of course, had he attempted to use them in a way they did not like, would have been instantly independent of him and inimical to him; though, had not the contrary been so positively asserted, we should have thought that passion would have been as communicable under such sympathetic circumstances, and after such a thorough blending of the physical nature, as the taste of wine. This writer treated a patient who had a tolerably exact knowledge ("eine ziemlich genaue Kenntniss") of the illness of her brother, who was at the distance from her of six hundred miles. This, however, does not equal an exploit of one of Wolfart's patients, who, on the 3d of August, 1811, at two o'clock in the afternoon, plainly saw her brother, who was staying on account of his health with a clergyman in the country, at the distance of some hundreds of miles. He was in a small field before the house at the time, walking *in the sun* with the clergyman; and they were both employed in looking for particular plants. She describes the dress of the latter, remarking that his coat was grey, and that he had on a very singularly shaped hat. Now, this 3d of August was an extremely hot day; and Wolfart was inclined to doubt the reality of the facts, from the circumstance *that the botanists did not confine themselves to the shade.* A letter was accordingly dispatched on the subject to the clergyman, who, in his answer, gave a full and particular account of the manner in which he and the brother of the somnambulist had been employed on the day in question, in the field before his house, in searching for the seed of the better sort of grasses, in order to improve his pasture; that, in order to pluck this seed just at the moment of maturity, they had been obliged to expose themselves to the heat of the sun at two o'clock in the day; that he had on a grey coat and a broad-brimmed hat, of which the brims slouched down, which he generally wore in rainy weather, but which he put on, on this occasion, to protect his face from the scorching heat. Whenever Wolfart directed the attention of this patient to her internal organs, she could see and describe them perfectly: this she did on the 4th of August, 1811, in the presence of Professor Grappengieser. She could hear Prof. Grappengieser whisper, but only when he was in contact with *her* professor. In the following month this woman saved Wolfart much anxiety and considerable expense in postage: his mother, who was many miles from Berlin, had a dangerous illness, and she described it to him day by day. She had never seen his mother, nor been where the latter resided; still she described her form, features, and whole appearance perfectly; and also the place she lived in. On the 7th of September she said, "I see her sitting in bed; her breathing is easier, but she lays her hand on her stomach as if she felt pain there." It was afterwards found that her affection had been principally one of the stomach, for which relief could only be procured by the application of the hand externally. On a subsequent day she said, "She is lying on the same bed, and is employed about something. It seems to me that there are several people going about the room, but I cannot recognize them. I think there is one sitting near the window. She is not so ill as she was; she appears to me to be like a person who does not leave her bed *because* she is better." When the old lady does manage to part with her pillow, the somnambulist announces the circumstance, and sees her walking about the room, leaning on another person's arm. We could not here, without occupying the space of this whole article, give any idea of the complexity and confusion of experiments which were tried, and of results which were obtained, in the case of this woman, in the presence of most respectable persons, generally mentioned by name, but must refer the curious reader to the original account of it published in the Ασκληπιειον, p. 1393. Let not the reader imagine that such stories as these are found only in German works on animal magnetism: we find even worse in the "Introduction" to the science by the Baron de Sennevoy, who at this moment is practising amongst the English aristocracy. In this the author gravely relates the case of a lady, who "appeared to a friend of hers in a house at a distance, and *cured her of the toothach.* She affirmed that it was her spiritual being, which, conducted by the soul of her mother, rendered this visit."* Neither time nor space exists for the somnambulist: she can embrace within her being the whole progress of events, and expand to the remotest distance. She can describe the past and divine the future.

Dr. Mertins, a physician practising at Berlin, asked one of his somnambulists whether magnetism had been used in the most ancient times, before the birth of Christ, and particularly by the Egyptians? She answered, *after a pause,*

"In a wide and sandy plain, where the air is pure and salubrious, at some distance from a large town, I see a temple, where physicians *or priests* are healing the sick. These are the Egyptians. The temple is of wood, rests on four pillars, and is entered by a flight of stone steps. It fronts the east. . . . . Now I enter a magnificent hall, on the middle of the dome of which there is a half moon and numerous stars. There are no windows, but round openings covered with green cloth, to diminish the intensity of the light. . . . Round along the wall are eighteen beds for invalids, or rather for sleepers, whom I see lying in them. The bedsteads as well as the pillows are stuffed with herbs. The beds are placed two and two, their heads towards the centre of the hall; a little nearer which is a circle of nine polished, shining, hollow, iron pillars, about three inches in diameter and three feet high. Each pillar stands on a triangular pedestal, which is filled with herbs; but the pillar itself contains quicksilver. The pillars are connected by a chain, and another chain intersects the circle which they form: along the latter sit patients, grasping it with one hand, with their backs towards the pillars, and holding with the other hand a ball with a cross projecting from it, three inches in diameter, hollow, and filled with herbs;† it looks like marble, but of what it is composed I cannot say. I see physicians with polished hollow iron rods, filled also with herbs, touching with them the parts affected of the patients. Two priests walk towards each other from the end of the chain, performing the treatment; and all the physicians keep touching the chain with balls like those of the patients, and shaking it. All the persons whom I see are clothed in white garments, and the priests wear a girdle around the body, which is buckled with a half moon in front, and on which are the figures of nine stars. The treatment of the patients is a religious rite, is only performed in the evening, and is best undertaken by moonlight. On the eastern side of the dome is a large opening, through which the moon pours her light into the interior of the building. There is another opening, which the priests make use of for watching the motions of the stars; for it is necessary to the perfection of their medical practice that they should be good astronomers and astrologers. They all live in celibacy, and choose the eldest amongst them for their chief or king, whose brow is encircled by a crown, and who sways in his hand the ball and staff, (from which, probably, the form of the modern regal

* Dupotet, p. 248.

† An engraving of both the temple and the ball are given in the original.

sceptre is derived.) . . . This temple lies near a large town, past which flows a river whose waters are of a reddish hue. At the present day," she added, "perhaps not a trace of this temple or city remains."

This somnambulist declared that, in the Vatican, there were many works on the ancient history and practice of magnetism; and one large book in particular, which she *saw*, and which a pious sage had translated from the hieroglyphics of the Egyptians into Greek.* Dr. Mertins hints to the Prussian government, that the magnetic establishment above described seemed to the somnambulist proper to be imitated on a large scale, wherever the state defrayed the expenses of such institutions. Wolfart remarks, that the whole article of Mertins is valuable, and not the less so because we lack historical proofs of the accuracy of this visionary description, and as to whether also the Egyptians were acquainted with quicksilver. For church-service (*Tempel-dienst*), says he, the somnambulist has here presented us with a great magnetic formula, which is surprisingly correct in its "polaric relations," which must be regarded as of incalculable efficacy, and of which the construction would do honour to the most sagacious and experienced magnetic practitioner. It furnishes a model, he continues, for all magnetic establishments on a large scale.† Not only does the magnetist claim as his the method of instruction, but he points to his somnambulist as an infallible source of all knowledge, historical and psychological, physiological and therapeutical, and even religious. Reason is superannuated; all research but folly; and every science but the *one* a contemptible and paltry delusion.

We now, with mingled feelings of wonder, melancholy, and disgust, approach the last chapter of the history of this grand modern instance of Credulity and Delusion.

The "Prophetess of Prevorst" first claims our attention, of whom her physician, Justinus Kerner, has published two volumes of more than 300 pages each, full of mystic details of her character and performances; all approved by such men as Schubert, Eschenmeyer, and Görres, who were and still are professors at German universities.

"Long before she was brought to me," says Kerner, "the whole earth, with its atmosphere, and all that was in and on it, mankind not excluded, existed for her no longer. She required more than one magnetiser,—more than the love, the zeal, the judgment which any one man possesses; she required what no mortal can bestow, another heaven, another air, and other nourishment than this world affords. She belonged to a world of spirits; she was half spirit herself; she belonged to the region beyond death, in which she already half existed. . . . . During the first years which she spent in the state which was not of this world, she might possibly have been withdrawn from it; but that was afterwards impossible. . . . . Still her residence here, at Weinsberg, (in Kerner's house, where she died,) was the most joy-

* The moon, this patient and her physician both state, had a particular influence upon her. "For seven years she was afflicted to madness by a nervous headach, of which she was at last cured by four months' magnetic treatment. When she experienced its attacks she went, at first driven by some instinctive compulsion, and afterwards voluntarily, to a mountain near her residence, on which she seated herself, and allowed the moon to shine upon her. She never did this without finding that it relieved her pains in the head."

† The article of Dr. Mertins' is the sixth in the first number of the second volume of Wolfart's Annals.

ful part of her spiritual life, and remains its brightest period, though many have sought to sully it with their poisonous spittle and ink. Her body clothed her spirit like a thin veil. She was small, of oriental features, and had the piercing eye of a prophet, the effect of whose glance was increased by her long and dark eyebrows and eyelashes. She was a flower of light, living on sunbeams."*

Eschenmeyer said of her, "Without apparent derangement of the vital functions, her life seemed to be only the glimmer of an expiring light. She was, as Kerner has admirably expressed it, a being who was continually dying, and whose life would have long since flown, had it not been retained by magnetic power. Her spirit seemed to be often separated from her soul, and the former frequently to dwell in other regions, whilst the latter was still bound to her body." (p. 60.) She said that she drew from the air "a particular nourishment, a living and vivifying principle." "Doubtless," adds Kerner, "the air which is allied to the sunbeams is the bearer of a stream of life from on high. She maintained, also, that there was a matter in the air, of which spirits availed themselves to render themselves visible and audible, which was prejudicial to her, but which was more observable on a fine than on a cloudy day." (p. 101.) "The spirit of all things, of which we in our ordinary condition have no perception, was perceptible to her, and operated upon her; more particularly the spirit of metals, herbs, men, and animals. All imponderable matters, even the different colours of the rays of light, produced upon her particular effects. To her the electrical fluid was visible and palpable." (p. 54.) "She perceived a weight distinct from all matter; she recognized a moral weight." (p. 79.) All kinds of substances, animal, mineral, vegetable, and fossil, were placed in contact with her, and the feelings which they produced duly recorded. The details of these experiments occupy thirty pages; they were repeated carefully, and varied in numerous ways. Whatever was given her to hold she took in her left hand, which she maintained was much more sensible than her right. Pressing it with her palm, she then directed her attention "to her left arm and side, then to the stomach, and thence generally to the lungs, brain, heart, &c., in order to discover what effect it produced. The smell of flint was agreeable to her. She tasted none of the stones, and still knew that fluor-spar was acid, and that muriate of barytes had a rough taste. Salt laid on her hand caused a flow of saliva; and copper, nausea and colic. Rock-crystal, placed on the pit of her stomach, produced rigidity of the whole body. 'Witherit' made her laugh, and fluor-spar always somnambulised her; though such different specimens of these fossils were used, that only a mineralogist could distinguish them as stones of the same species." (p. 74.) Grapes, of the thousand different sorts which are cultivated on the banks of the German rivers, were put into her hand, and all with different results. The feelings which they all excited in her corresponded exactly to the effects which followed the wines made from them: this is duly attested by Mr. Göritz, a *gourmet*, of Stuttgard. (p. 81.)

"The bone of an elk threw her into a sort of epileptic fit; the horn of a chamois cured her of spasms. The nipple of a mare produced a remarkable effect upon her brain: had she epilepsy, she said, it would be of great service to her. It figured

* Die Seherin von Prevorst, p. 59.

frequently in the prescriptions she ordered for herself. Powdered, for instance, she had it rubbed into her spine, as a remedy for its weakness; she also used it to smell of when she was faint. It might be introduced into the materia medica. This animal substance seems to contain a quantity of ammonia, and has a very peculiar odour; in some trifling degree similar to that of castor. I only find it mentioned in Paracelsus, who used it in an ointment for the plague." . . . "The tooth of a mammoth caused her a feeling of sluggishness, which was probably a consequence of the slothfulness and dulness natural to that animal. . . . A spider's web, rolled into a ball, produced a prickly feeling in the hands, and a restlessness in the whole body: might it not be of benefit in delirium tremens? Glow-worms threw her into a magnetic sleep." (p. 89 *et seq.*)

"Whilst the prophetess was at Weinsberg, the sun had only the following effect upon her: When she lay towards the occident, she menstruated continually; when towards the south, regularly. When she was not regular, she had only to turn towards the west. Once in sleep she described the cause of all this, but it was not recorded. The red ray of light rendered by degrees her whole body cataleptically rigid; but she recovered by the contact of barytes. A violet ray somnambulised her; which is remarkable, since it makes iron magnetic, and is said to promote the growth of plants. . . . The moon seemed to have no influence on her, unless she gazed on it: then it called forth feelings of sadness, coldness, and shivering. Looking at it caused her also to menstruate, but only, she maintained, through the sun, and only as long as she looked at it. She said, 'If the moon acted upon me like the sun, I should be far worse than I am.' (p. 98.) . . . "Music somnambulised her: in order to make her cheerful, she was accustomed to request me to magnetise the water she drank by playing the jew's harp. She used to say in sleep, 'Set the water in my glass in motion by seven strokes of that steel music.' If she drank water thus magnetised, without even knowing that it had been so, she was generally constrained involuntarily to sing. . . . The sonorous vibrations of a glass after it has been struck, she seemed to hear much longer than other people.* . . . . The eye of many men somnambulised her. She frequently said, that there lies in the depths of the human eye a spiritual spark, which she would call the mirror of the soul; and through this the image of the external object, which falls on the retina upside down, is again turned round," &c. (p. 102 *et seq.*)

If she laid a magnetised rod on her right eye, and then gazed on any object, she saw it magnified. On looking thus at the moon, she said, that, on its left side, its inhabitants were much employed in building, and were more happy than those of the right side. She drew from the eyes and lips, and the fingers of other and stronger persons, sometimes without their feeling it, a *pabulum vitæ*. Numbers of patients were sent to her by the most skilful physicians, who had tried their skill on them in vain; and she performed the most astonishing cures. On the 5th of September, 1837, Kerner put in her hand a piece of ribbon, on which was written the name of a woman who was ill, but the nature of whose illness was unknown both to her and to him: the ribbon had been worn by the invalid, and its contact produced in the prophetess the malady of the latter,—"violent sickness, pain in the bone of the left foot, oppression of the chest, and a peculiar irritation of the uvula." (p. 167.)

She had a particular language and system of numbers and calculation, of which long descriptions, explanations, diagrams, and engravings are given. These had a particular reference to different phases of her being, which she called her solar and vital circles, of which long interpretations are published by the celebrated Görres, professor at the university of

* On magnetism by sound; Kerner quotes Mesmer, and the example of the prophet Elisha, (2 Kings, iii. 15.)

Munich, one of the most remarkable men in Germany, both as a political and religious character, and as a man of action. The phrase *bianachli,* which she only knew how to pronounce in her vital circle, and which she translated with great repugnance on her solar circle, means in Hebrew "I am in sighs." The following phrases we give as examples of her language: *Optini pogu,* go to sleep; *o minio pachadastin,* I am asleep; *mi lo arato,* I rest; *posi anin cotta,* the ring is full.

The Prophetess of Prevorst saved her brother's life, by warning him that a man was about to attempt it. She said, "He who is planning my brother's murder is a person of twenty-six years of age, and he does not live in the same village as my brother. I see only a few houses in the place where he is;—you turn to the left to go to them. There he is in a house two stories high. But it is now enough; and I thank thee, my God, that I know that my brother is saved." Then she prayed in a low voice.

Spirits in vast numbers visited her; but she did not seek their acquaintance; they forced themselves upon her.

"I often see," she said, "many with whom I do not come into contact; then, again, others who come to me, with whom I speak, and who often spend months in my company.* I see them even when I am awake, and they often wake me out of my sleep. I hear and see other things at the same time; but I cannot turn my eyes from them, for I am, as it were, in magnetic *rapport* with them. They look like thin clouds; but are not transparent, though they at first seem so; still I never saw one which cast a shadow. Their form is like that which they had during life, only colourless and grey; their clothing is also similar to that which they wore when alive, but it is also colourless, as if made of cloud. The brighter and better spirits, however, have on a long garment, hanging in folds, with a girdle round the waist. The expression of their features is generally solemn and sad. Their eyes are bright, like a fire. None of them, that I ever saw, had hair on the head. They make noises, particularly to excite the attention of those who have not the gift of seeing them: these noises consist of sounds in the air, sometimes sudden and sharp, and producing a shock; sometimes musical; at others resembling the rustling of paper, the falling of sand, the rolling of a ball, &c. They can carry heavy substances, overturn tables, knock plates together in the rack, &c. The better spirits are brighter than the bad ones, and their voice is not so strong. Many, particularly the darker ones, when I uttered words of religious consolation, sucked them, as it were, in, and I saw them become brighter and lighter in consequence; but I was rendered weaker. Most of the spirits who come to me are in the lowest regions of the spiritual world, which are situated in our atmosphere: they were the grovelling ones of this world, or such as did not die in the Christian faith, or else such as in expiring clung to some earthly thought, which now weighs them down. In these inferior regions the spirits are still exposed to the temptations of the devil. I once asked a spirit whether children grew after death. The answer was 'Yes; the soul gradually expands its vest, until it is as large as it would have become on earth.' I cannot effect the salvation of these spirits; I am only their mediator; I pray ardently with them, and so lead them by degrees to the great Saviour of the world; but it costs an infinity of trouble before such a soul turns again to the Lord." (vol. ii. p. 10 *et seq.*)

Herr von Meyer, the author of "Pages from Prevorst" and "Pages for higher Truth," says, in the latter work, alluding to these spirits, "The soul (of the dead) is often far from being spiritual. On the con-

* We may here premise, what will shortly fully appear, that there are a host of witnesses living, ready to attest the "facts" mentioned in this work; a host of persons ready to swear that they, too, saw or heard the ghosts.

trary, it is frequently poor in spirit, foolish, full of errors as to the means of bettering its condition; and, in its deeds and desires, is often as absurd and laughable as any madman." Eschenmeyer says, "The heavenly spirits doubtless are ideally beautiful; truth there assumes her snow-white robe, and virtue receives her crown; but with ghosts of earth, how different!—blear-eyed lust may stare through their hollow mask, vice becomes a monster, and crime shrouds itself in the black steam of hell. The wedding-garment of heaven is only sent to those who have been bidden to the marriage of the Lamb; and the night-dress of earth is left the sinful, who must stand without, and to whom the Lord says "I know you not."

On the 31st of December, 1825, whilst the prophetess was singing a psalm in the house of her father at Oberstenfeld,* a noise was suddenly heard, as if something heavy had fallen to the ground. Nothing was seen; but, when she went to bed, her lamp was observed and heard to flicker about, and all at once the cloudy form of an old knight stood before her. In her alarm, she called to the maid-servant, who was near her, to come and sleep in her bed: the latter attempted to bring with her some of her bedclothes, but these were taken from her and kept back by an invisible hand. The ghost then disappeared. The next night, as the clock struck twelve, when her brother was in her room, it again appeared, shook her bedstead, and said to her, "If thou wilt not go with me, I will throw thee out of the window." She answered, "In the name of Jesus, do it." He vanished, reappeared, and exclaimed, "I'll throw thee into the deep cellar." She answered, "In the name of Jesus, do it." He then vanished again; but returned again in a few minutes, and threatened to stab her. But she said, "That thou hast no power to do." He now once more melted away, and did not repeat his visit for three nights. When he again appeared, he said to her, "Thou must go with me, for I have hidden an inkstand. Under the sandbox is some writing and a few coins. This stand I must give thee—then I shall have rest." She said, "I cannot go with thee: this inkstand cannot render thee happy." In subsequent conversations she directed him to the word of God, and reminded him that there is only one Saviour, taught him to pray, and prayed for hours with him, kneeling by her side. He confessed to her that he had murdered his brother, and that he was a member of the Weiler family of Lichtenberg. On the seventh night this apparition told her that the hour of his liberation was near, and thanked her for having brought him to his Saviour. His whole figure was now much more friendly and bright. On a sudden appeared seven of his children, all white, shining, and joyful: they were grown up; they formed a circle round him, and sang in exquisite melody. The knight and the prophetess joined them, and the latter fell into a sleep, in which she continued singing for some time: when she awoke, she found the knight still by her side, who wanted to mark her hand in remembrance of him, and who would not quit her until the ghost of her grandmother stepped between them, and drove him off. (vol. ii. p. 80.)

Shortly afterwards the ghost of another murderer appeared to her:

* It was not till afterwards that she left her husband, a Mr. Hauffe, by whom she had children, to live with Kerner—and his wife.

this was a short, dark, wrinkled figure, who wore a cowl. He told her to treat him like a child, and instruct him in the rudiments of religion. He haunted the house, and amused himself by perplexing its inhabitants in every possible way, so that her father determined to leave it. All kinds of noises were heard, and in every direction; and, if any person ran to see what was the matter, they instantly broke out in other places. Sometimes the prophetess ran away from the spirit, and one day, in trying to escape him, she tumbled over the threshold. He sought to lift her up, but could not. Then she felt on her right arm a hand, and saw a white figure, which raised her from the ground.

She took a walk in the middle of the day once, with her parents, her brother, and a female friend, to Bottwar. As they were returning, and had reached the garden of the abbey at Oberstenfeld, the clock struck seven, and the ghost (who always chose this hour to appear at) instantly stood before her. She was compelled to run with it at an incredible rate, and her friends heard it as it dragged her along, flapping in the air and against the walls of the houses. She went with it into a deserted kitchen, and they there prayed together. One evening she walked to Gronau; and, as she was not back by seven, the ghost came to fetch her. She now flew rather than ran with it home; her feet did not touch the ground; it was impossible for any person to follow her. The ghost swept on before her, and now and then relaxed his pace, and breathed out the words "Pray for me! pray for me!" They went again to the kitchen, where he folded his hands, knelt, and prayed with her calmly. Every time, after praying, the spirit uttered some short sentence, of which, says Kerner, few unluckily have been preserved; they are as follows: "Now a sun rises within me!" or "Now the sun shines within me."

It is impossible to describe the solemnity and devout conviction with which the author of, and the contributors to, the work before us speak of such scenes as the foregoing. Its motto is, "I thank thee, Father of heaven and earth! that thou hast hidden those things from the wise and prudent, and revealed them unto babes." After relating one of the miraculous performances of the prophetess, Kerner exclaims, "Recognize here, O thoughtful reader, the power of spiritual sympathy, prayer, and childlike faith!" On this exclamation Eschenmeyer observes, "Ah! my friend, they will recognize it not; they have not even a distant idea of what spiritual sympathy is; they cannot feel what prayer or what childlike faith consists in." (vol. i. p. 179.) Kerner declares that he visited this patient at least three thousand times, and that she cannot have deceived him; (vol. ii. p. 34:) as to the assertion that she was cracked, he allows that she was "as cracked as Plato was." (vol. ii. p. 38.) Indeed, all her biographers and commentators are evidently deeply impressed with the belief that they are dealing with irrefragable and awful truths. They betray no personal feelings; their whole nature is subdued to the grandeur of the cause, whose furtherance, as they imagine, has been imposed upon them from on high. They are as much the slaves of the somnambulist as she is theirs. Kerner's anger is never roused except when she is attacked: of his own reputation he does not appear to have the slightest care. We know nothing that they can possibly gain by their labours; for they claim no merit to themselves, and their pursuits are certainly most unprofitable in every sense: they confess that they are often in helpless ignorance until enlightened by their somnambulist. Of the sincerity of all of them no doubt has ever been expressed in Germany; of their learning and standing in society we need not again remind our readers, though we may remark, in addition, that two of them, at any rate, Schubert and Görres, have a European reputation from their scientific and literary labours. The work itself is written with a mystic fervour perfectly in accordance with the subject; it is very ill arranged, and interlarded with quotations from old chronicles, from Jacob Behmen, Paracelsus, Herr von Meyer's "Hades, or a Theory of the Science of Ghosts," the Philadelphia Journal of the Medical and Physical Sciences, the Times newspaper, and a thousand other sources; but the style is generally good, where not too mystical. The first volume is principally occupied with an account of the magnetic condition of the somnambulist; the second with a detailed history of her ghostly experience. It is often, particularly in the psychico-theological parts, utterly unintelligible to any reader. In some parts which we have not touched upon, we find accounts of contemporary wizards, of amulets, sympathetic powders, charms, conjurations: indeed, the work may be entitled "An Essay towards the Restoration of Witchcraft; with an account of it as a Science, and Directions for practising it as an Art." It appears that the only alteration which this ancient profession undergoes at the hands of its revivers is that, instead of being heretical, it is now a most thoroughly Christian practice. From the bright effect produced by the prophetess on the dark spirits, we find that it has also changed from a *black* into a *white* art. That the former, however, in spite of her piety, would have been burnt alive in the middle ages, there cannot be the slightest doubt; for Kerner is at pains to assure us that she was so light that she could not, by any means short of a millstone, be kept under water.

The consummation of animal magnetism is, then, that the most illustrious somnambulist that ever lived was a witch, who departed this life on the 5th of August, 1829, and who now lies buried at Löwenstein, in the kingdom of Würtemberg. There is nothing more instructive in the history of the prophetess than the fact that, no sooner was it rumoured that she was haunted, than ghosts began to appear throughout all the kingdom of Wurtemberg.

The history of her life at Weinsberg is nothing but a continuation of ghost-scenes. She brought her whole stock of spirits with her, and kept most of them by her until her decease. The business she transacted with each is described under the head of "Facts:" we have "First Fact at Weinsberg," a section twenty-six pages long; "Second Fact," nine pages; "Fourth," sixty pages, and so on. We can give no idea of the scrupulous minuteness, multiplicity, and extravagance of the details: generally, a considerable number of other persons are in some way mixed up with the "facts." The parson of the parish was waited on by the sprites who came to settle at Weinsberg with her; and he has duly attested this, after giving manifold details in a formal declaration, signed "Weinsberg, June 5th, 1827. C. W. Hermann, clergyman." (vol. ii. p. 190.) This reverend gentleman put questions to some of the visitants of the prophetess, as to what religion they were of; as to whether they knew the mother of our Lord; as to whether the Virgin Mary stood in a more intimate relation to her exalted Son than any other happy Spirit, &c.? One of the answers he obtained was, "I know the mother of our Lord somewhat better than thou dost." (vol. ii. p. 190.) The "Fifth Fact" relates to an eccentric ghost, who came drest in a Tyrolese hat and spurs, and who one day, in the presence of Kerner and her sister, pulled off her boots, and carried them to the window; the next night he called again, bringing an ugly old female spirit with him; and, as soon as they entered the room, they put out the light. (vol. ii. p. 213.) "I clearly understand," says Kerner, "that ghosts are not seen by the eye, but by magnetic waking of the inner man." The "Eleventh Fact" is a declaration signed W. F. Pfleiderer, Heilbronn, October 20th, 1828; and is an account of how, when he visited the prophetess, he was hugged by the ghost of his schoolmaster, who happened to be staying with her at the time. (vol. ii. p. 264.) Of one, at any rate, of her ghostly visiters a lithograph has been published: this was a woman in an ancient costume, with a human heart in her hand: she herself made a drawing of her. (p. 275.) In February, 1829, the prophetess was consulted by "a robust, florid, lively woman," Mrs. Herlinger, whose husband was landlord of the *Eagle* at Grossgartach, near Heilbronn, and who was troubled by the importunities of a ghost without a head. Kerner reflected much as to who this individual, whose postures and proceedings were sometimes very indecorous, could possibly be, but did not succeed in settling the point to his satisfaction. The prophetess was true to magnetism to the last, and even longer; for, after she had been dead for some time, and her cheeks had become cold and stiff, her mother made three magnetic passes over her face, and she again opened her eyes and moved her lips.

The work terminates with some poetry by Kerner to her memory. The last page but one contains the results of a post-mortem examination of her body by Dr. Off, of Löwenstein, who never saw a more "beautifully formed" brain than hers, nor a healthier one. Preceding the details of her death is a letter from a clergyman, seventy years old, Mr. Seeger, of Altburg, congratulating Kerner on the new evidence of the immortality of the soul, and of the power of Christianity, which the prophetess has afforded. "I stand," he says, "on the threshold of eternity, and welcome every means of confirming my faith and hope."

We find in Wolfart's Annals (vol. v. part i. p. 74,) an account of a woman whose performances eclipse those of even the prophetess.

> "Her somnambulic sleep commenced with the apparition of an evil spirit; then a good one overshadowed her, with whom she appeared to converse, and who took her with him through the sun and moon into the mansions of eternity. Then he appeared to her at an immense distance. Her physician, and those who saw her during her converse with this spirit, describe her features as being like those of one transfigured, whom no pencil could draw."

"Die Fingerzeige Gottes," one of the works quoted at the head of this article, was published, in 1838, by Brockhaus of Leipsic: it is the work of a lady, Madame de S., who, whilst under treatment by a physician, somnambulised her maid-servant, and by means of the latter conversed with the Almighty. The book consists exclusively of their dialogues, which are set down in the most ordinary phraseology, are on the most trifling and absurd topics, and are held, indeed, in every respect, in the most familiar and every-day tone. The following are specimens:

"*Question of Madame de S.* Shall I soon be ripe enough for immediate *clairvoyance?*

"*Answer of the girl.* He says, 'Not directly. Thou shouldst not write and think so much. Thou shouldst eat more nourishing food and plenty of raw eggs.'" (p. 22.)

"*Quest.* Tell him how I have enjoyed to-day, out of my window, the beauties of his works.

"*Ans.* Nature is everywhere beautiful." (p. 30.)

"*Mad. de S.* I cannot conceive, Lina, (the girl's name,) how it is that the Creator, Preserver, and Ruler, our dear Father, is not everywhere felt and adored.

"*Lina.* He says, in his goodness, 'I thank thee for that.'

"*Mad. de S.* Oh, God! Lina, tell him quickly that his love for me is all the gratitude I require." (p. 48.)

"*Mad. de S.* Is there a devil?

"*Lina.* There is no devil and no hell." (p. 49.)

"*Mad. de S.* I am afraid I speak, without thinking of it, too familiarly to him, as if I were talking to my mother.

"*Lina.* 'Am I not also thy Father?'

"*Mad. de S.* Tell him not to think too highly of me; not to make me too confident.

"*Lina.* He says, 'I will not lead thee astray.'" (p. 51.)

"*Mad. de S.* Could we illuminate our room every evening?

"*Lina.* 'My child may amuse herself in this way whenever she pleases.'

"*Mad. de S.* Yes; but thy child is not rich enough to do it.

"*Lina.* 'Child! child! Thou sayest that thou art not rich, and thou hast, whilst still on earth, half won the kingdom of heaven.'" (p. 117.)

"*Mad. de S.* Art thou sure it is not an angel who speaks to us? Is it the Father?

"*Lina.* He says, he is sure it is he himself." (p. 128.)

We have left ourselves little room to treat of the cures performed by animal magnetism. The most universal medicines do not pretend to be of avail in bringing man into the world, or restoring him to it when dead; they do not boast of being especially efficacious in arresting the progress of disorganization; they are not advertised as a remedy in the acutest forms of enteritis, in phlegmasia dolens, in pestiferous carbuncle, and in hydrophobia; but all these claims are put boldly forth by animal magnetists.* According to this sect, all diagnosis is superfluous, pathology is an absurdity, and therapeutics what every man has at his fingers' ends.

It is with reluctance that we turn to the English page of this strange eventful history. The censure which we have not wantonly bestowed even on remote offenders, cannot be dealt without pain on those nearer to ourselves, and whose estimable characters and actual services to science plead in extenuation of apparent error and unaccountable temporary delusion.

The English boast of a reputation for practical good sense, and sound philosophy. As a nation, we are certainly neither apt to indulge in extravagant pursuits of our own creation, nor to adopt those of our neighbours; but still there are individuals amongst us, it would seem,

* See Wolfart's Annals, vol. i. part i. p. 138, and p. 183; and Bork's Heilungen durch Magnetismus, p. 21 and 84.

who can be emulators to a certain extent, of the exploits of the continental philosophers, whose labours we have faithfully commemorated above. It might have been supposed that animal magnetism would here assume a more rational shape than on the continent; but the fact is, in even the trifling extent to which comparatively it has been enabled to develop itself here, it has given promise of greater contradiction and absurdities than it has anywhere else presented. In no other country has it been prosecuted with a more blind empiricism, or with such apparent ignorance of the progress which it has elsewhere made. Our magnetisers have began *de novo*, not, it would appear, because they doubted the truth of the results which others had arrived at in the science, but because they never took the trouble to enquire into the nature of these results. The history of their favorite science they only seem to have studied in the Reports of French commissions, and in a few other similar, unsatisfactory documents: of the hundred authors who have written in German on the subject, not one appears to have been studied by their English brethren.

The most noted magnetisers, during the last two years in London, have been Dr. Elliotson, Mr. Mayo, Dr. Macreight, Dr. Sigmond, and M. Dupotet. Dr. Elliotson seems to have entertained no theory of the art, but to have confined himself simply to the research of facts; and the nature of those which he has encountered, has been such as to lead him to adopt views partaking, more or less, of the character of all the continental schools. His system, as far as we can learn from some of the conclusions to which he has arrived, will prove to be eclectic: he combines the doctrines of Mesmer with those of the somnambulisers. Though himself a somnambuliser, he allows "that it is not necessary that a *sensible* effect should be produced in cases in which nervous diseases are successfully treated by the process of magnetism."* In this respect he approaches the school of Ziermann and Andresse, but his patients, the O'Keys present phenomena, which can only be classed with those observed in the case of the Prophetess of Prevorst. One of them was asked, while in somnambulism, "whether she could lift eighty-four pounds." She replied, that "the negro,"—a spirit, which she says constantly attends her, and whom she consults on various occasions, told her, "she could lift eighty, but it would hurt her ribs."† Though, amongst the facts ascertained by Dr. Elliotson, there are many from which every German theorist might draw proofs of the truth of his particular doctrine; though some confirm the views of Kluge, and some those of Kerner; there are others that tend to refute every theory which has ever been constructed on the subject. Such are the dangers of eclecticism! Such a fact as the following, demolishes at once the foundation of all the continental systems of magnetism. "Dr. Elliotson coming in front of the patient, caused her to magnetise herself, by desiring her to make bows to her face," with one hand. After half a dozen of these passes she fell asleep! Hitherto, all professors of this singular science, however they may have differed on minor or even major points, have agreed that its essential foundation is the action of one organism upon another. But here we have a person, at once som-

* Lancet, 1837-38, p. 378. † Ibid. p. 382.

nambuliser and somnambulist, magnetising herself. This discovery at once renders obsolete the four hundred German volumes on magnetism. Another fact is, not merely generally but magnetically absurd; not merely a stumbling-block to common sense and ordinary criticism, but to the very principles of the magnetisers themselves. " Mr. Wood was led to try the effects of magnetising the reflection of Jane O'Key in a looking-glass. Being told to look at herself in an unframed glass, two passes were made at her image, when she fell into the same condition of sleep as when magnetised personally. The glass being held obliquely, the same result followed, though her attention was drawn elsewhere."* This experiment was varied in several ways, and was always attended with equally singular results. Jane O'Key mixed with her somnambulic conversation some snatches of an unknown tongue. A board was placed before her eyes, upon which she sang, " Sound the loud timbrel," and on coming to the line, "Jehovah shall triumph," she said to the board, " Will you triumph, you dirty beast. I'm sure you won't, Misce crutis, crece croo," words which were unintelligible.† This unknown tongue evidently bears a greater resemblance to dog-latin than to those Coptic and Hebrew languages, from which the sublime Prophetess of Prevorst derived her somnambulic voice. But though of a far lower degree than the latter heroine of transcendentalism, and though, in her case, somnambulism did not seem, as with Kluge's impregnably virtuous patients, to elevate and purify the character, but rather the contrary, O'Key has still well earned the appellation of the " Prophetess of St. Pancras;" for we learn from Earl Stanhope, " that Dr. Elliotson had written to him to say, that O'Key had foretold the occurrence of a severe rheumatic pain eighty-four hours before, and the disease actually came on at that time."‡

We shall make no comments on the new doctrines which have been broached on the subject of magnetism in this country, and which we now proceed to describe in the words of their professors, leaving it to the reader to learn, from their internal contradictions, and from their incompatibility with each other, and with those of continental sages, the forlorn and hopeless state of this science of delusion. Dr. Macreight is of opinion, that " Mesmerism is not a cure for a disease, but a cure for particular persons, diseases of any kind occurring in whom this agent would cure."§ Dr. Sigmond says, " The art seems to me to consist in obliging the individual again to inspire, by the nostril, the carbon he has already expired, whilst the currents of air caused by the extended fingers produce some effect upon the facial nerves, thus inducing the eye-lids to fall down."|| Mr. Mayo is certain, that " by *looking* upon a *mesmerisable* body, you may so mesmerise it, that *another mesmerisable substance laid upon it shall from it be mesmerised* sufficiently to produce decided mesmeric effects upon patients susceptible of this peculiar agency."¶ " I think," says he, " that the phenomena of prevision and transposition of sensation naturally lead to the supposition, that they result from the workings of a spiritual nature, in a certain

* Lancet, 1837-38, p. 402. † Ibid. p. 287. ‡ Ibid. p. 370.

§ Ibid. p. 369. || Ibid. p. 389. ¶ Letter in the Medical Gazette.

independence of those bodily organs to which it is normally closely tied and bound; from the mind being in part dislocated and displaced from her corporeal tenement, holding on with misplaced attributes to unaccustomed points and corners of the frame."

The patients to whom Mesmerism has in this country been principally indebted for the development of its most singular phenomena are two girls, Jane and Elizabeth O'Key, the former of whom, previous to her admission to the North London Hospital, and treatment by Dr. Elliotson, was under the care of Dr. Theophilus Thomson, who states, " that he attended her originally for phrenitis, which was followed by epilectic fits. Depletion and calomel for a time relieved her, but the fits eventually returned, and resisted the treatment employed. He found her on one occasion with the senses, vision included, apparently suspended; this lasted for a day or two. On another occasion he found her in a state of ' classical' delirium, in which she had an extraordinary memory of the names of diseases, and the remedies which are employed for them. All these effects had occurred independently of animal magnetism,"* to which state accordingly, to use the expression of Bertrand, she was very easily "*façonnée.*" Mr. Wakley says, however, that Jane appears, on a cursory examination to be but a tame copy of her sister Elizabeth, "who is a genius in her line." This is betrayed by her dark, piercing eye, her wonderful performances, and the power which she exercises over all who have come much in contact with her. Her improvisations at the mesmeric sittings, the witticisms, the sarcasms, the snatches of song, were not unfrequently worthy of the licensed fool of the old comedy: the audience was often amused, when the jokes derived their raciness neither from ribaldry, profanity, nor obscenity. Her very impudence was *naif*. The talent which she possesses in greatest perfection is imitation. In the course of some experiments (at Mr. Wakley's house) she took occasion to descant on " affected young ladies," gave several examples of the character delicately drawn, and, having worked up the imagination of the audience, concluded with a characteristic interrogative,—" Now what would I do with such a *fine* young lady? Why kick her—to be sure."† He thinks, that as an actress, she would make the fortune of a theatrical manager; and states, that she formerly figured as a prophetess and performer in the unknown tongue at the Rev. E. Irving's chapel.

The exhibitions and experiments made at the North London Hospital, and reported in the Lancet, equal in marvellous absurdity and incredible credulity anything which has ever been reported of continental magnetism. On Sunday, June 3, Elizabeth O'Key was lying dressed on her bed; Dr. Elliotson, Mr. Wood, Mr. H. Mayo, the Rev. Mr. J——, Mr. J. Thomson, and the various patients and nurses were in the ward. "At a quarter to five, Mr. Wood, with some difficulty, woke her by rubbing the eyebrows and pressing the palms: she rose up in bed, and presented a peculiar expression of countenance; one of fierceness and resolution. Her features were rigid, firmly set, and sharpened with intensity of feeling. She might be likened to an eagle, wounded in the wing, and brought to the ground, eager to tear some enemy, but imbecile from want of

* Lancet, 1837-38, p. 379. † Ibid. p. 873.

motive power. A piece of paper was thrown to her, she tore it into fragments with her hands and mouth. 'O'Key, O'Key,' Dr. Elliotson said. 'Leave me alone, you villain, do!' she exclaimed, darting towards him with the ferocity of a tiger. (Her voice was full, sepulchral, and resonant, having the depth and force of a powerful adult voice,) 'you —! get away.' .... Dr. Elliotson tried to mesmerise her. The process had no result. Mr. Wood,—'Where's the negro, O'Key?' O'Key, 'You — fool be off.'" Details of this description too filthy to sully our pages withal, fill no less than three columns. In triviality, repetition, and evident credulity of the observers, they remind us strongly of the cases and conversations given in Wolfart's Annals; much more so than we should at first be led to anticipate.

The following scene may be taken as a fair specimen of the exhibitions at the North London Hospital, which were attended by nnmbers of the aristocracy, and by several individuals of scientific eminence.

"Elizabeth O'Key was put to sleep with a single pass. Jane, on seeing her, laughed; and, hugging her, said, 'Oh! you silly thing, you shouldn't live that way.' Dr. Elliotson remarked, as every one was struck with her peculiar manners, 'that she was one of the best-hearted girls in the world.' Some gentleman sat on the ground to rest, 'Oh! don't sit that way,' she said, 'Your name isn't Norval, if you sit in that poor place.' Dr. Elliotson, 'Look up there,' (at the crowd.) O'Key, 'Oh! what a many white ones. Why, where the d—l did you all come from?' (*Great laughter.*) As she spoke, a slight pass of the hand from some visitor behind her stupified her; and, as she stood, Mr. Wood also behind her, to the right, drew his hand, pointed towards her side, at a yard distance, gradually away from her. The process turned her round from a front position to an oblique one. He continued the motion with his hand, and the girl fell asleep, and dropped to the floor. She was awoke by blowing on her eyes, and on recovering her legs began to skip and sing,—

'I went into a tailor's shop,
To buy a suit of clothes,
But where the money came from,
G—— A—— knows.'

Immense laughter followed this distich, and from that she proceeded to sing—

'Malbrook she went to be shaved
And the barber he cut her white chin,'

when her *volatility* being too great for any other experiments, Dr. Elliotson said, 'he must stupify her,' which a single pass of one finger, before her face, effected in a moment; the girl passing from the state of excessive merriment to that of cataleptic rigidity."*

We need not, we presume, dwell on the experiments by which Mr. Wakley proved that the pretended magnetic phenomena observed in the O'Keys were developed by these girls at will, or at any rate were not the result of any mesmeric influence or agent, but occurred as well without as with the manipulations which are supposed to communicate the latter. Dr. Elliotson (and here again he adopted principles novel and adverse to the systems of his continental brethren, holding that the nature of its medium essentially modifies the effects of the Mesmeric agent,) asserted that magnetised nickel produced remarkable and peculiar symptoms, and that lead produced none at all; but on pieces

* Lancet, 1837-38.

of both these metals being successively applied to the hands of Elizabeth O'Key, no effects at all were produced at first, and afterwards the expected phenomena made their appearance on her hand being rubbed with a piece of lead and a farthing. In the same way mesmerised water and mesmerised sovereigns produced no effect upon either of the sisters, though they both of them became sometimes "fixed" or "stupified," by the contact of an unmagnetised sovereign, or by sipping unmagnetised water.

Over the other details of London magnetic experience we willingly draw a veil. Our object has not been to give expression to our feelings, but to present to the reader's consideration an historical record, which may be reflected upon with some benefit. Neither would we be so far influenced by the impostures occasionally practised under the name of magnetism, as wholly to deny that some of the phenomena, from time to time produced by all aspirers to the art, seem to result either from some principle heretofore unknown and not yet correctly designated, or from some modification of recognized principles in the animal economy which cannot yet be accurately limited or defined. The whole of man's existence is too mysterious, and he is surrounded by too many things utterly beyond his comprehension, to justify an obstinate disbelief of things hard to be understood. In the constant attempts of the human intellect to penetrate the thick curtain that hangs all around it, doubtless some transitory glimpses of hidden truths are now and then accorded to quick intellects and peculiar organizations; and there is ever much more in heaven and earth "than is taught in our philosophy." The temporal guides of man, however, are his senses and his reason; and when he lays claim to a wisdom and to powers which are incapable of being made palpable to the one or explicable to the other, although we may not presume to say that he cannot possibly be right, he must expect that we make very diligent use of our own senses and our own reason in the investigation of his evidence; and industriously endeavour to untwist the double chain of truth and fancy, which he would fain twine round our puzzled understandings.

